# Optimizing anisotropic margins in single‐isocenter multiple brain metastases radiosurgery using regressor strategies: A multi‐institutional validation study

**DOI:** 10.1002/acm2.70249

**Published:** 2025-08-31

**Authors:** José Alejandro Rojas‐López, Miguel Ángel Chesta, Francisco Tamarit, Néstor Daniel Vacca Campos, Mariana Hernández Bojórquez, Maximiliano Musso, Alexis Dimitriadis, Carlos Daniel Venencia

**Affiliations:** ^1^ Facultad de Matemática Astronomía, Física y Computación Universidad Nacional de Córdoba Córdoba Argentina; ^2^ Hospital Angeles Puebla Puebla México; ^3^ Instituto de Física Enrique Gaviola (Universidad Nacional de Córdoba and CONICET) Córdoba Argentina; ^4^ Queen Square Radiosurgery Centre National Hospital for Neurology and Neurosurgery London UK; ^5^ Fundación Valle del Lili, Cali Valle del Cauca Colombia; ^6^ American British Cowdray Medical Center Artificios S/N Ciudad de México, CDMX México; ^7^ Instituto Zunino Córdoba Argentina

**Keywords:** healthy brain, multiple‐metastases, PTV margins, radiosurgery, single‐isocenter

## Abstract

**Purpose:**

To explore a comprehensive method for assigning anisotropic margins in single‐isocenter multiple‐metastasis radiosurgery (SRS), utilizing regressor strategies with geometric, dosimetric, and mechanical information. Such margins may reduce the volume of the irradiated brain without compromising the dose to lesions, by considering additional treatment variables.

**Methods:**

First, the impact of slice thickness on the margin was investigated on an anthropomorphic phantom for different lesion volumes. In addition, for clinical cases, the anisotropic margins were assigned using two methods: by geometric margin criterion (GMC) based on clinical protocols and optimized margin criterion (OMC) based on regressor strategies. To evaluate the plans (456 metastases in total), we reported the Paddick conformity, gradient, and efficiency indices, monitor units, the volume of brain receiving at least 12 Gy (V12), and risk of radionecrosis by two radiobiological models (Flickinger and normal tissue control probability models).

**Results:**

An overestimation of volumes was related (absolute difference of 0.13 ± 0.18 cm^3^) to a larger slice thickness and smaller target volumes. The OMC demonstrated an overall reduction (20%) of the target volumes. OMC also showed improvement in gradient index and monitor units compared to GMC. V12 showed a statistically significant decrease when OMC was used compared to GMC, ranging from 5% to 41% across all three institutions (*p* = 0.045). The radiobiological models pointed out that the risk of radionecrosis was lower than 10%, and there was no statistically significant difference between GMC and OMC (*p* = 0.151).

**Conclusion:**

While the feasibility and potential dosimetric benefits of anisotropic margins in SRS—such as improvements in the gradient index and V12—were observed, the magnitude of these differences may be modest in terms of clinical impact. The results suggest that anisotropic margins can be integrated into SRS workflows and may offer advantages in specific scenarios, particularly when minimizing brain dose is a priority.

## 1 INTRODUCTION

Stereotactic radiosurgery (SRS) is a well‐established and growing treatment modality for brain metastases.^1^, ^2^, ^3^ In recent years, single‐isocenter multiple brain metastases SRS (SIMM‐SRS) has demonstrated advantages compared to the multiple‐isocenter technique (MIMM‐SRS).^4^, ^5^ Furthermore, given that SRS demands high‐dose delivery, high‐dose gradient, and sub‐millimeter precision, it is imperative to thoroughly consider all sources of uncertainty, especially in single‐fraction SIMM‐SRS. Nevertheless, sources of uncertainty that must be considered when determining margins for planning target volume (PTV) in SRS may be underestimated.

In other words, reports 50 and 91 of the International Commission on Radiation Units and Measurements (ICRU), explain that the PTV is a geometric concept that considers the combination of systematic and random treatment errors.^6^, ^7^ For example, the total workflow uncertainty of frameless SRS using the Gamma Knife is 1.3 ± 0.5 mm,^8^ indicating that an additional margin may be needed in this setting. Notably, no significant differences in control or toxicity were seen between patients treated with and without margins.^9^ Nevertheless, recent technical guidelines from the International Society of Radiosurgery for the treatment of multiple brain metastases, reported that PTV margin expansion depends on the quality assurance results for each machine, while no consensus regarding the maximum margin acceptable was agreed between the authors.^10^ Thus, it is important to comprehensively reduce margins from a geometric and dosimetric perspective. Therefore, the use of robust, fast, and accurate tools that take into account different parameters for decision‐making in margins appear promising.

Moreover, PTV margin reduction is not commonly practiced. However, it has shown good local control when anisotropic margins, based on the position of the gross tumor volume (GTV) concerning organs at risk, are considered.^11^ In SRS, the use of anisotropic margins is a concept that has been explored to a limited extent based solely on the image guidance system through the van Herk formula,^12^, ^13^ while other available data on the subject focused on conventional radiotherapy for extracranial anatomical sites^14^ or animal studies.^15^ Therefore, it is prudent to conduct more research in this area, with novel tools such as machine learning to consider different SRS parameters in the assignment of anisotropic margins.

Machine learning is uncommon in the practice of SRS, but is commonly used in research tasks such as the delineation of targets,^16^ co‐registration,^17^ clinical prognosis,^18^ or to differentiate radiation necrosis from disease progression.^19^ Recently, genetic algorithms were used to obtain the maximum displacement produced by rotations/translations in SIMM‐SRS.^20^ However, the reliability of any machine learning model's confidence in its predictions is critical for high‐risk applications, especially in deciding the margins in SRS. The synthetic models in machine learning are never 100% accurate in their predictions.^21^

While genetic algorithms have been used to model rotational uncertainties, no existing method integrates geometric, dosimetric, and mechanical parameters through supervised learning. Our approach uniquely combines regression strategies with anisotropic margin optimization to address the balance between the comprehensive PTV margin assessment while simultaneously limiting irradiation of healthy brain tissue. The application of regressor strategies to determine the necessary margins for each GTV seems reasonable and effective. These strategies could be objective and robust, providing automatic approximations that reduce decision times while providing quantitative metrics that allow evaluating their performance in SIMM‐SRS.

To address the above problem, we developed a computational Python‐based open‐access method for regressor strategies in order to optimize PTV margins. This was conducted by performing a closed (validation stage using a batch of the training data from one institution) and open validation (test stage using multicentric data) in three institutions. The parameters considered in the analysis included the plan quality indices and the dose received in the brain on SIMM‐SRS treatments with and without optimized margins.

## 2 METHODS

Before investigating the impact of anisotropic margins on plan quality (taking different sources of uncertainty into account), we studied the impact of the slice thickness of computed tomographic (CT) images on PTV reconstruction. Subsequently, the variables of multileaf collimator (MLC), lesion volume and diameter, dose cluster formation (high dose bridging), lesion number and location, distance from the isocenter, and position uncertainty due to imaging/mechanical issues, which are associated with mechanical uncertainties of the linear accelerator were considered.

### 2.1 Impact of CT slice thickness

In a Somatom goUp unit (Siemens Healthineers AG, Erlangen, Germany), a set of CT images was acquired with a slice thickness of 0.6, 1, and 2 mm of a PseudoPatient head phantom^22^ with nine metallic fiducials placed in plastic spherical holders (6, 10, and 15 mm) randomly distributed. In the treatment planning system (TPS), the images were imported and the GTVs were contoured. The contouring was performed following the shape of the plastic spherical holders. An isotropic 1 mm margin was then added to generate PTVs. To analyze the impact of contouring and margin creation, the relative differences between physical and contoured volumes were evaluated.

### 2.2 Plan selection

Anonymized plans from three institutions were used in this study (Table 1). These were planned as single‐fraction treatments using dynamic conformal arcs on Elements (Brainlab Elements, Brainlab AG, München, Germany) with an energy of 6 MV with flattening filter, and dose prescriptions of 18–24 Gy utilizing SIMM‐SRS and the Brainlab pencil beam algorithm.^23^ Institutional ethical clearance was granted ensuring patient anonymity.

**TABLE 1:** Description of the plans used by three institutions.

Training and closed validation		
Parameter	Institution A, Training	Institution A, Closed validation
Total number of patients	50	7
Total number of brain metastases	316	48
Mean number of metastases per patient	6±6 [2, 40]	7±5 [2, 14]
Mean GTV size (cm)	1.24±0.96 [0.09, 6.26]	1.00±0.53 [0.43, 3.16]
Mean GTV volume (cm^3^)	1.33±4.31 [0.01, 18.80]	0.46±0.87 [0.02, 5.07]
Mean distance to isocenter (cm)	4.6±1.6 [0.9, 10.1]	4.7±1.7 [1.6, 10.1]
Dose prescription criterion	21 Gy, with a PTV coverage of 99%	
PTV‐margin criterion	Isotropic margin. If the GTV is located less than 50 mm from the isocenter, a margin of 0.5 mm.	
	If the GTV is located more than 50 mm from the isocenter or its volume is smaller than 0.1 cm^3^, a margin of 1 mm.	

Open validation		
	Institution B	Institution C
Total number of patients	8	5
Total number of brain metastases	50	42
Mean number of metastases per patients	6±4 [3, 15]	8±6 [4, 19]
Mean GTV size (cm)	1.17±0.89 [0.29, 4.85]	1.05±1.13 [0.27, 6.70]
Mean GTV volume (cm^3^)	1.16±3.33 [0.01, 19.91]	0.40±0.79 [0.01, 3.81]
Mean distance to isocenter (cm)	4.2±1.8 [0.7, 8.6]	4.7±1.4 [1.8, 9.5]
Dose prescription criterion	21 Gy, with a PTV coverage of 99%	15, 18, or 20–25 Gy depending on PTV size, with a PTV coverage of 98%
PTV‐margin criterion	Isotropic margin. If the GTV is located less than 50 mm from the isocenter, a margin of 0.5 mm.	Isotropic margin. 1 mm to each brain metastasis
	If the GTV is located more than 50 mm from the isocenter or its volume is smaller than 0.1 cm^3^, a margin of 1 mm.	

The reported values correspond to arithmetic mean ± 1 standard deviation [min, max].

Magnetic resonance images (MRIs) with 1‐mm slice thickness were co‐registered with CT images on the TPS. To account for the volume reconstruction in the GTVs and PTVs, the different CT slice thicknesses used across institutions, 0.6‐mm (two institutions) or 1‐mm (one institution) slice thickness images were used to create the contours. All fusions and organ at risk (OAR) contours were performed automatically in the TPS using the Elements Anatomical Mapping tool, which contoured OARs by registering the CT images to a synthetic full‐body tissue model including more than 100 cranial structures; then OARs were inspected and approved by the radiation oncologist.

Geometric distortion correction, by the Elements Distortion Correction Cranial tool,^24^ was applied in all cases where it was considered clinically relevant. The GTV was delineated on the MRI by the radiation oncologist. The PTVs were created following the isotropic margin criteria in Table 1 using the contouring tools in the TPS. PTV dose prescriptions followed international guidelines.^25^, ^26^ The minimum acceptable plan quality was 98% coverage of the target volume (TV) by the prescription isodose, and a Paddick Conformity Index (PCI) higher than 0.5.

### 2.3 PTV margin assignment

PTV margins were assigned in two ways. In one case, the geometric margin criterion was followed as described in Table 1.^4^, ^27^, ^28^ In the other case, an optimized margin criterion (OMC)^29^ was applied through geometric, mechanical, and dosimetric variables. The detailed description is outlined in the Supporting Information. It is summarized in the following steps:

1. First approximation of isotropic margin based on set‐up uncertainties: The first approach to the margin is based on intra‐fractional set‐up uncertainties, by calculating the displacement produced by 0.2°/0.2 mm rotations/translations, in the 6 degrees of freedom, through a genetic algorithm.
2. Correction of isotropic margin based on MLC: If the margin assigned to a lesion showed a PTV size lower than the size of two leaves of the MLC, the margin was increased until it reached at least the value of the width of two leaves. This was done to ensure that all metastases were covered by at least two leaves.
3. Correction of isotropic margin based on small GTV volumes: If the PTV volume is less than 0.1 cm^3^ (approximately 3 mm diameter), we increased the PTV‐GTV margin to achieve a PTV volume of 0.1 cm^3^ (for a lesion of 1 mm diameter, it implies a 1‐mm PTV margin).
4. Isotropic margin prediction by regressor models: Then, to build the regressor strategies, we introduced as input the total number of metastases, GTV size (diameter), GTV volume, location, distance to isocenter, and the dose cluster formation. The CT slice thickness is not considered at this point. The output is a discrete value between 0.1 and 1.0 mm.
5. Final tuning by anisotropic margin considering CT slice thickness: The PTV margins assigned were anisotropic, opting for the conservative approach to picking the biggest from the margin options: In the anteroposterior and left‐right direction, the margin is the output obtained from the regressor strategy described in point 5, and for the cranio‐caudal direction the margin adopted the largest value between the predicted output from the regressor and the CT slice thickness.
6. PTV anisotropic margin assignment for dose calculation: These margins are assigned to the GTVs, and the plans were recalculated.

### 2.4 Supervised regressor strategies

Four different supervised regressors were developed ranging from low to high complexity. The models were coded in Python v.3.10 in a public repository and included the linear model (LM), multilayer perceptron (MLP), random forest regressor (RFR), and extreme gradient boosting regressor (XGB).^30^ Additionally, we used the models with feature selection (wFS) by mutual information to eliminate the variables that showed dependency on each other.

The best parameters for the optimization of each model were obtained by the grid search technique and the k‐fold technique.^31^ During the k‐fold cross‐validation process, five splits were used. For the MLP, the optimized parameters included the hidden layer sizes, the learning rate, and the strength of L2 regularization. The activation function was fixed as the rectified linear unit, and the solver was fixed as the stochastic gradient‐based optimizer. For the RFR, the optimized parameters were the number of trees, the maximum depth of the trees, the minimum number of samples required to split an internal node, the minimum number of samples required to be at a leaf node, and the number of features considered when determining the best split. For the XGB model, the optimized parameters included the learning rate, the maximum tree depth for base learners, and the minimum sum of instance weight needed. The booster was fixed as the gbtree function, and the sampling method was fixed to randomly select training instances uniformly.

The training and closed validation were performed as described in the Supporting Information. The inputs must be input as a 1‐D vector consisting of six parameters: the total number of metastases, the brain lobe where each metastasis is located, the GTV volume in cubic centimeters, the GTV size in cm, the distance to isocenter in millimeters, and the dose cluster formation.

The dose cluster formation is assigned as a binary number. To consider if there is a cluster, we calculated the Euclidean distance between the center of mass of two lesions. If the Euclidean distance is lower than the sum of the radius of the two lesions plus 6 mm, there is a dose cluster formation. The value of 6 mm was based on the dose gradient index per lesion, considering that the ideal fall‐off from the prescription isodose to the isodose for half the prescription is 16.6% per millimeter.^32^

Margin predictions were evaluated by calculating values for the accuracy, the mean absolute error (MAE), and the cross‐validation score (CVS). Accuracy was determined by the number of cases, *N*, that matched the expected values as shown in Equation 1. The MAE score was calculated as the average of the absolute differences between the expected, $y_{j}$, and predicted, ${\hat{y}}_{j}$, values as shown in Equation 2. The expected and predicted values correspond to the displacement produced by rotations and translations applying the 0.2°/0.2 mm tolerances for intrafraction uncertainties,^20^ and the margin obtained by strategies, respectively. These values were set into a new plan to recalculate. These plans were evaluated by the radiation oncologist and medical physicist to consider if they were clinically acceptable.

During the validation stage, plans were recalculated using the predicted margins. Evaluation is as described in the following subsection.

(1) $$\mathrm{Accuracy}=\frac{\mathrm{Number}\;\mathrm{of}\;\mathrm{corrected}\;\mathrm{predictions}}{\mathrm{Total}\;\mathrm{number}\;\mathrm{of}\;\mathrm{predictions}\;\mathrm{made}}\;,$$

(2) $$MAE=\frac{1}{N}\;\sum_{j=1}^{N}\left|y_{j}-{\hat{y}}_{j}\right|,$$

The use of augmentation techniques to enhance the diversity of data is critical during training to achieve robust and generalizable models.^.^ ^33^, ^34^ In order to prevent the overfitting of the regression model, a data augmentation strategy was used by creating more data points (2160 values) based on the existing data by noise injection to input variables. We used a zero‐mean Gaussian kernel and random noise was added to existing individual data by the sum of the input and a random normal between 0 and 1, as described in Equation 3:

(3) $$x_{j,new}=x_{j}+\left[\mu +\sigma \sqrt{2}\;erf^{-1}\left(2p-1\right)\right],$$

where $x_{j}$ is the original input value, $x_{j,new}$ is the generated value from the data augmentation strategy, p is a random number taken from a uniform distribution between 0 and 1, μ is the mean (to reduce any bias it was considered equal to 0), σ is the standard deviation considered as the X% of $x_{j}$.

In order to describe the reliability and prediction uncertainty of the model predictions, the confidence interval was obtained for each model using bootstrapping as a resampling technique in Python. Through this method, we generated a distribution of 5000 sample estimations, rather than a single point estimate. The confidence interval was calculated by the 95% of the MAE and the *R* ^2^ (coefficient of determination) regression score using the 2.5th and 97.5th percentiles of the bootstrap estimates.

Institutions B and C used the open code to predict the margins in the validation stage. They reoptimized the plans, preserving the initial objectives and goals, with these predicted margins by OMC, and the medical physicist with the radiation oncologist assessed if the plans were clinically acceptable.

### 2.5 Dosimetric evaluation

The recalculated plans (OMC) were compared with the original ones (GMC) by assessing the differences in quality indices for the PTVs and the monitor units (MU). The dosimetric changes were analyzed by evaluating the differences in PCI, gradient index (GI), volume of brain tissue receiving at least 12 Gy (V12), and the other indices such as D99 and efficiency index (EI) for GMC and OMC plans, in that order.

The EI combines the qualities of conformity, gradient, and a high mean dose to the target into a single value.^35^ It is calculated as the ratio of the integral dose of the TV to the integral dose of the volume encompassed by the prescription isodose (PIV). Considering multiple targets with different dose prescriptions, $EI_{12Gy}$ is calculated as in Equation 4:

(4) $$EI_{12Gy}=\frac{\sum_{i}D_{mean,TV_{i}}\times TV_{i}}{D_{mean,PIV_{12Gy}}\times PIV_{12Gy}}\;$$

where $D_{mean,TV_{i}}$ is the mean dose of the i‐th TV ($TV_{i}$), $D_{mean,PIV_{12Gy}}$ is the mean dose of the volume encompassed by 12 Gy ($PIV_{12Gy}$).

In addition, one of the main predictors of radionecrosis is the V12.^25^, ^36^, ^37^ The V12 predicted risks of post‐treatment changes suggestive of necrosis.^25^, ^36^, ^38^ In this work, for V12 evaluation, the healthy brain was defined as the volume of the whole brain minus the volumes of the GTVs and the brainstem. To describe this effect, the risk of necrosis was evaluated using two methods. The first method adopted a normal tissue complication probability (NTCP) model,^25^ as shown in Equation 5:

(5) $$NTCP=\frac{exp\left(4\gamma_{50}\left(\frac{V_{X}}{V_{X},50}-1\right)\right)}{1+exp\left(4\gamma_{50}\left(\frac{V_{X}}{V_{X},50}-1\right)\right)}\;,$$

where *V*_x_ is the volume receiving greater than or equal to x Gy dose, *V*_x,50 _= 63.2 cm^3^ is the volume corresponding to 50% risk of any necrosis with γ_50_ = 0.87 the slope parameter.^25^ Moreover, we analyzed values of V12 considering the number of brain metastases in the plans in two cases, where there were < 10 and ≥10 metastases.

The second method considered a model used to predict permanent symptomatic post‐radiosurgery injury^39^ employing a risk‐location score based on the location of the lesions in the brain (the model is derived from an arteriovenous malformation population, but is being applied here to brain metastasis). The estimated probability (P) of developing a permanent symptomatic post‐radiosurgery injury (necrosis) from the final logistic regression model was calculated using Equation 6 ^39^:

(6) $$\begin{array}{cc} & P\left(necrosis\right)\\ & =\frac{exp\left(-7.8713+0.7506*SPIE+0.0734*V12\right)}{1+exp\left(-7.8713+0.7506*SPIE+0.0734*V12\right)}\;, \end{array}$$

Thus, the plan quality was assessed using the quality indices (PCI > 0.5, 3 < GI < 6, V12 < 10–15 cm^3^). For statistical analysis, a paired *t*‐test for normally distributed data was performed in Python v.3.10 with a *p*‐value equal to 0.05 to establish statistically significant differences between the quality indices.

## 3 RESULTS

### 3.1 Impact of CT‐slice thickness

**FIGURE 1:**
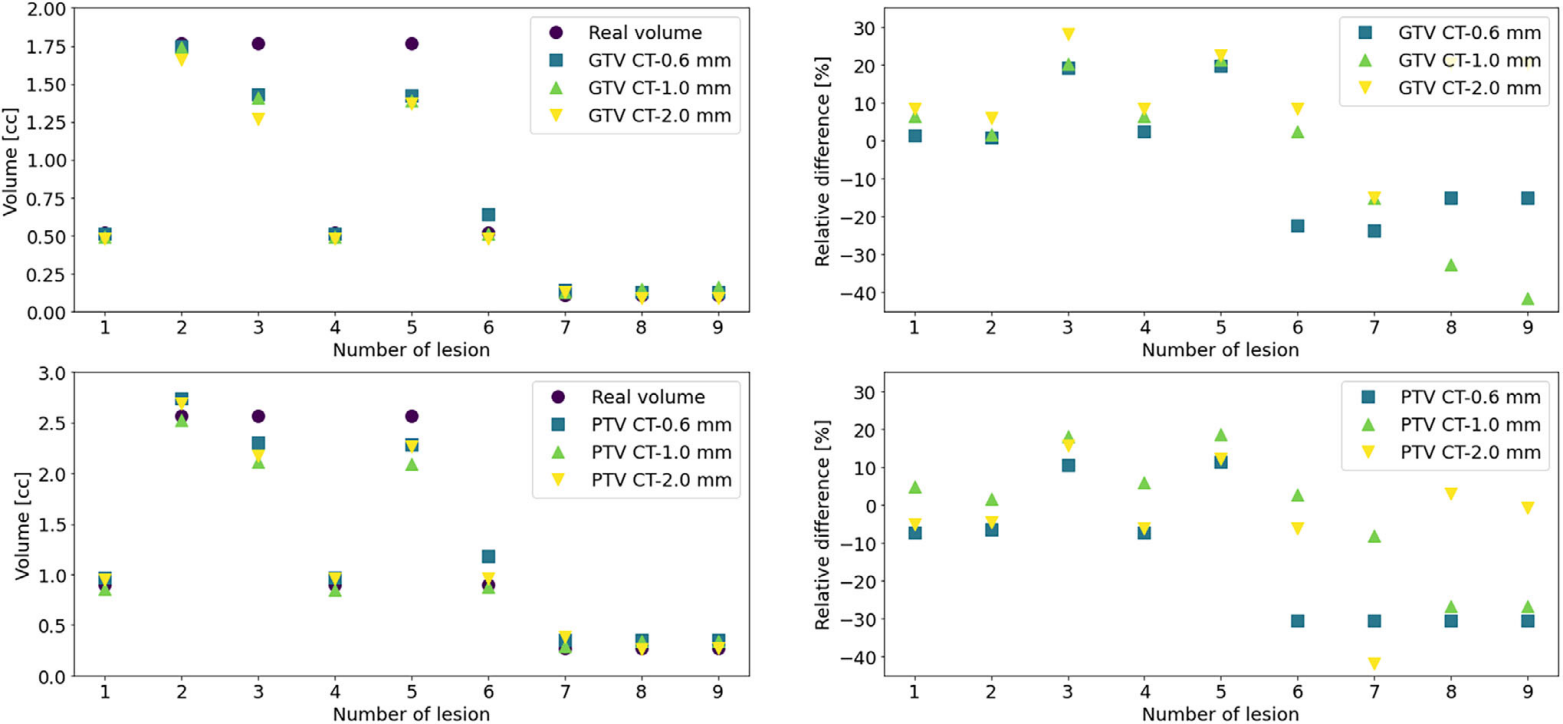
Upper left) GTVs volume vs CT‐slice thickness. Lower left) PTV volume with 1.0 mm of margin. Upper right) Relative differences for the GTVs. Lower right) Relative differences for the PTVs.

The impact of CT‐slice thickness resulted in larger differences (absolute difference of 0.13 ± 0.18 cm^3^) for CT images with a larger slice thickness and smaller GTV volumes, as shown in Figure 1. Moreover, after the creation of PTV margins, the differences in the volumes were pronounced by up to 30% for small targets and thinner CT slice thickness. Table 2 shows the mean values and standard deviation of the GTV and PTV (GTV plus 1 mm margin) volumes with regard to CT slice thickness and GTV size. The p‐values were calculated by the U Mann‐Whitney test, showing statistically significant differences for small lesions (*p* < 0.05). These results demonstrated the necessity to establish PTV margins considering the impact on the volume reconstruction, especially for small lesions, which has been incorporated in the OMC prediction for the margin in the cranio‐caudal direction.

**TABLE 2:** Mean and one standard deviation of the reconstructed volumes for the GTVs and PTVs regarding the CT slice thickness and the size of the lesion.

	Size of the lesion (mm)	CT slice thickness (mm)	Reconstructed volume (cm^3^)	*p*
GTV	3	0.6	0.13 ± 0.01	0.059
		1.0	0.15 ± 0.02	0.063
		2.0	0.10 ± 0.02	0.637
	5	0.6	0.55 ± 0.07	0.637
		1.0	0.49 ± 0.01	0.059
		2.0	0.48 ± 0.00	0.046
	10	0.6	1.53 ± 0.18	0.063
		1.0	1.51 ± 0.19	0.063
		2.0	1.43 ± 0.20	0.063
PTV (GTV+ 1mm)	3	0.6	0.35 ± 0.00	0.046
		1.0	0.32 ± 0.02	0.059
		2.0	0.30 ± 0.06	1.00
	5	0.6	1.04 ± 0.12	0.059
		1.0	0.86 ± 0.01	0.063
		2.0	0.95 ± 0.01	0.059
	10	0.6	2.44 ± 0.26	0.642
		1.0	2.24 ± 0.26	0.063
		2.0	2.37 ± 0.27	0.642

### 3.2 Comparison of regressor strategies

**FIGURE 2:**
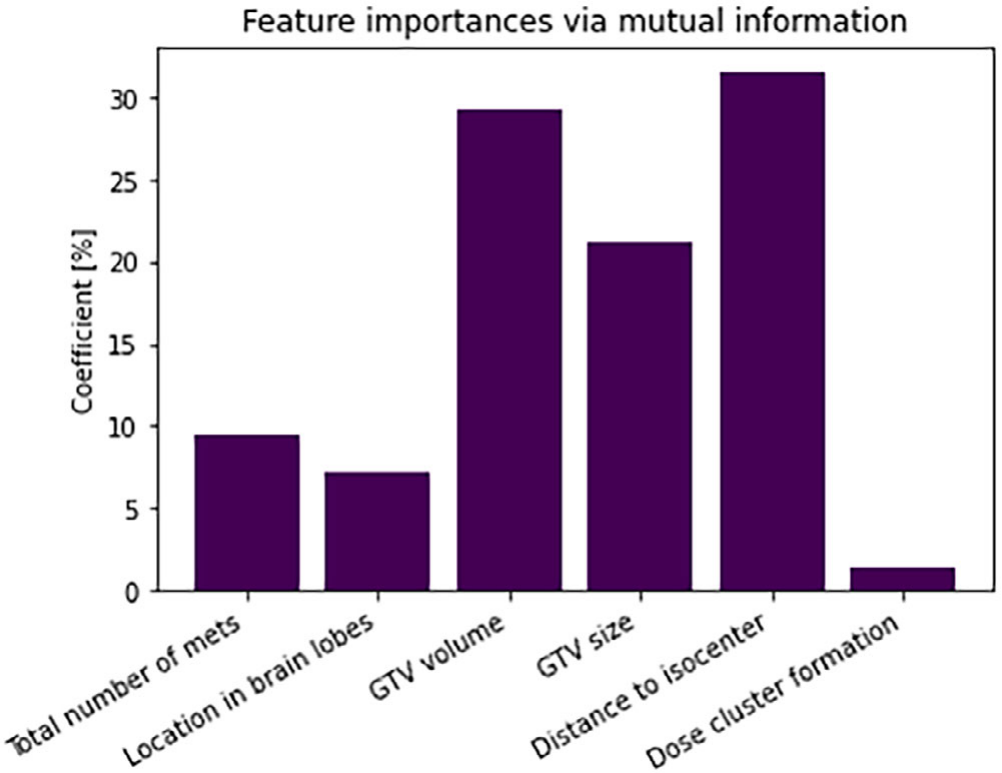
Feature selection for the input parameters of the regressor models.

To understand the relative importance of input parameters within the models, an analysis of the models with feature selection (wFS) was performed. Figure 2 shows the feature importance of each parameter through mutual information. Mutual information revealed that geometric features such as the distance to isocenter (29.8%), GTV volume (27.9%), GTV size (21.4%), and the total number of metastases (10.4%) are the most predictive features for model training.

**FIGURE 3:**
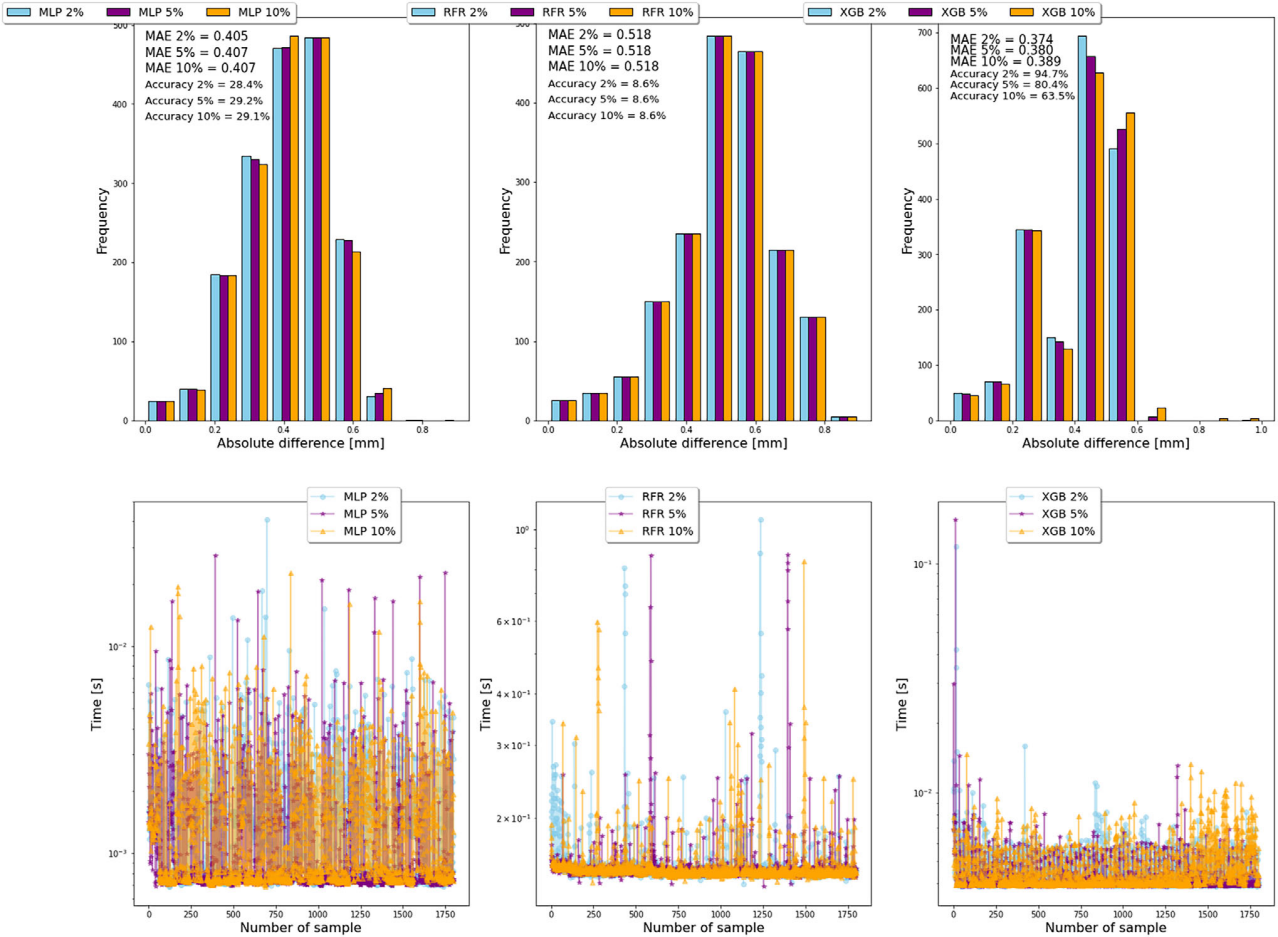
Gaussian noise injection in terms of MAE, accuracy, and computational time for MLP, RFR, and XGB in terms of the percentage of the standard deviation considered as the X% of input values.

The overfitting by Gaussian noise injection in the training stage was evaluated through the MAE, accuracy, and computational time. Figure 3 shows that 2% of the input values reduced the possible bias in the predictions based on the MAE (0.347), accuracy (94.7%), and computational time (0.0046 s per sample), mainly for XGB. The value of 2% deviates from the original input values up to 10%, as shown in Supporting Information.

**FIGURE 4:**
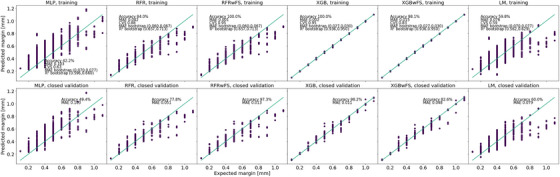
Regression strategies results for the expected versus the predicted PTV margins.

In addition, accuracy, MAE, CVS, and bootstrap distributions for all regressor strategies are reported in Figure 4. The results concerning the models to prevent the overfitting are reported in Supporting Information. Figure 4 illustrates the association between the expected and the predicted values for the training and the closed validation datasets. The highest CVS is reached by the XGB strategy (0.95), the accuracy is the highest (98.2%), and the MAE is the lowest (0.011). Thus, XGB was used to predict the margins during validation.

### 3.3 Geometric versus optimized PTV margin

**FIGURE 5:**
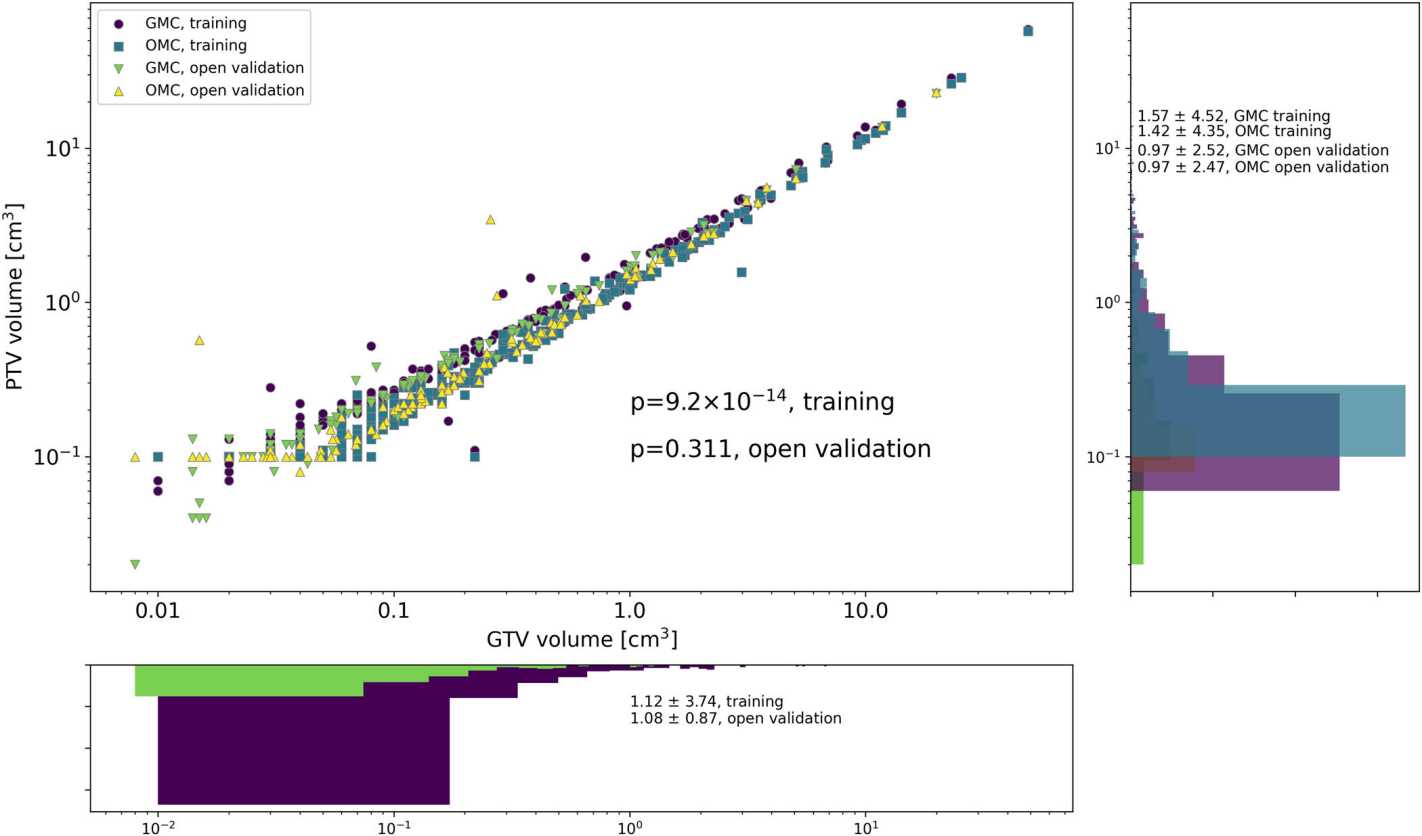
PTV versus GTV volumes by GMC and OMC for the training and open validation. The histograms showed the distribution of the volumes for all cases.

Figure 5 shows the relation between GTV and PTV by the use of GMC and OMC, statistical differences were calculated for training (*p* = 9$\times 10^{-14}$), and no differences were found for open validation (*p* = 0.311). The mean value of the PTV was reduced in training and open validation as shown in Figure 5 comparing the OMC to GMC. This PTV‐volume reduction is associated with the use of margins in the range from 0.1 to 1.0 considering the variables described in this work. Additional correlations for the multiple inputs for the XGB are reported in Supporting Information.

### 3.4 Dosimetric comparison and statistical analysis

In addition, the quality indices for the plans considered in the validation for the three institutions are presented in Table 3. The statistical analysis indicates that margin assessments using OMC resulted in similar mean values of PCI, EI and MU (*p* > 0.05). For D99, and GI, statistically significant improvement was observed (*p* < 0.05). In general, the mean value for these indices improves for OMC, that is, GI and MU are lower.

**TABLE 3:** Comparison of the dosimetric indices for the plans used in the validation for the three institutions (A, B, and C) using the geometric and optimized margin criteria (GMC and OMC, respectively).

	GMC					OMC				
Institution	D99 (Gy)	PCI (a.u.)	GI (a.u.)	EI (a.u.)	MU	D99 (Gy)	PCI (a.u.)	GI (a.u.)	EI (a.u.)	MU
A	19.8 ± 0.6	0.81 ± 0.05	5.13 ± 1.30	0.37 ± 0.07	9296 ± 3998	19.8 ± 0.5	0.81 ± 0.06	4.77 ± 0.99	0.38 ± 0.07	8623 ± 4231
B	20.4 ± 0.7	0.73 ± 0.10	4.78 ± 1.42	0.39 ± 0.08	12712 ± 5681	20.4 ± 0.7	0.74 ± 0.10	4.61 ± 1.22	0.29 ± 0.13	11288 ± 4223
C	20.1 ± 3.1	0.64 ± 0.07	6.24 ± 0.07	0.24 ± 0.07	13503 ± 3373	19.9 ± 2.7	0.69 ± 0.09	5.72 ± 1.12	0.28 ± 0.09	10527 ± 1935
*p*	–	–	–	–	–	0.014	0.189	8.5$\times 10^{-5}$	0.460	0.065
*t* statistic	–	–	–	–	–	2.471	−1.320	4.147	0.761	1.957
Critical value of *t*	–	–	–	–	–	1.977	1.980	1.991	1.770	2.093
Pearson correlation coefficient	–	–	–	–	–	0.948	0.781	0.857	0.432	0.680

### 3.5 Risk of radionecrosis (V12 and radiobiological models)

Table 4 shows a detailed description of the impact on mean V12. It results in an improvement of the mean value of the V12 compared to the original V12 (*p* = 0.045).

**TABLE 4:** V12 for healthy brain reported for the three institutions during open validation using GMC and OMC.

Institution	GMC V12 (cm^3^)	OMC V12 (cm^3^)
A	2.17 ± 2.06	2.05 ± 1.94
B	5.82 ± 11.04	4.70 ± 8.95
C	2.30 ± 5.57	1.35 ± 2.06
*p*	–	0.045
*t* statistic	–	2.013
Critical value of *t*	–	1.977
Pearson correlation coefficient	–	0.871

**FIGURE 6:**
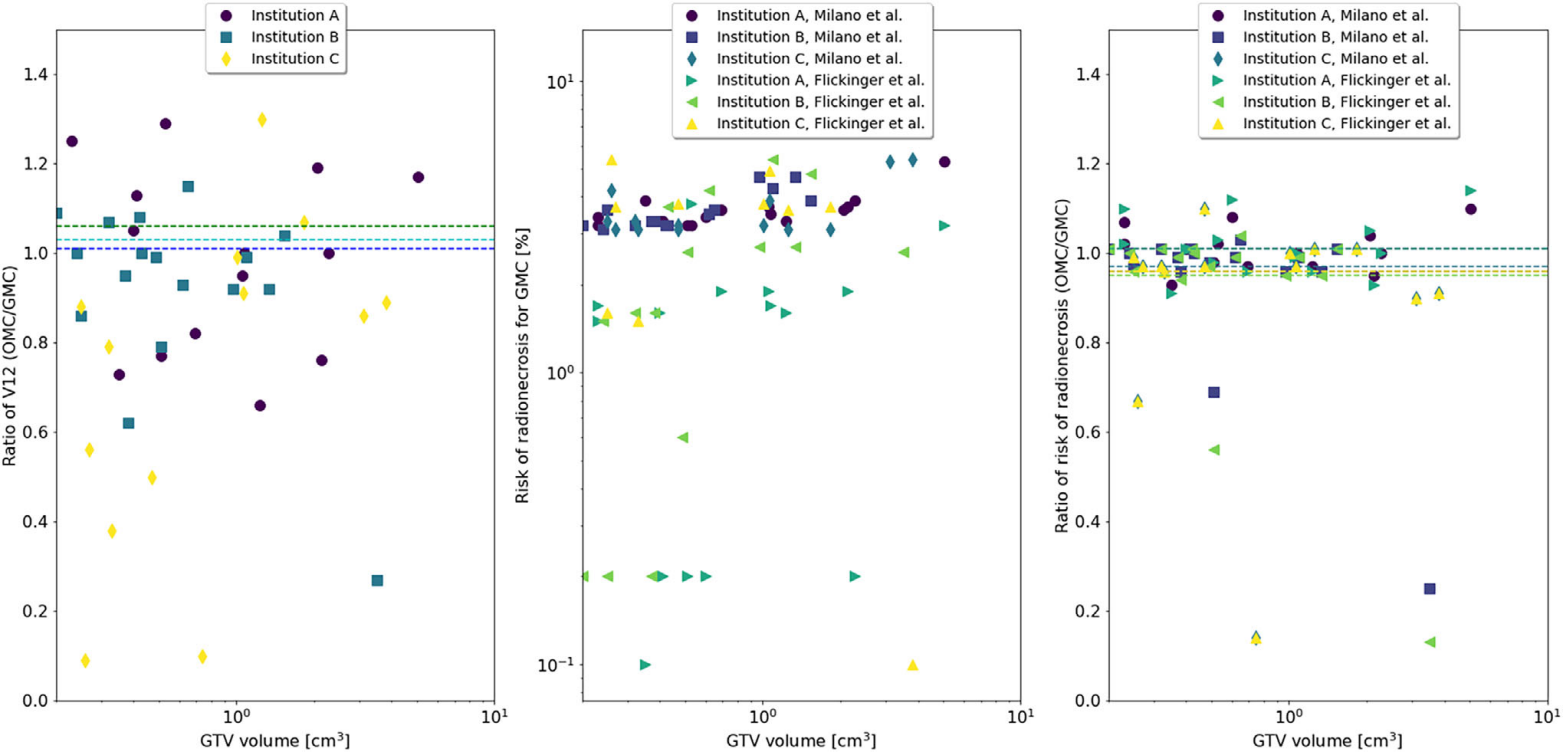
Left: Ratio of V12 for the three institutions using GMC and OMC versus GTV size. Dotted lines correspond to mean values. Center: NTCP values for GMC. Right: Ratio of the radiobiological models.

Additionally, the ratio of V12 and the two methods of assessing the risk for radionecrosis (Milano and Flickinger) for the brain, considering GMC and OMC, is reported in Figure 6. Thus, we reported that for most cases, no matter the number of treated brain metastases, V12 and the risk for radionecrosis for OMC were within acceptable values. For most of the cases, the risk for radionecrosis obtained for the two models was lower than 10% and no statistically significant differences were reported. The mean values for Milano's model for GMC and OMC was 3.9% and 3.7%, respectively (*p* = 0.133). For the Flickinger's model, the mean values were 1.6% and 1.4%, respectively (*p* = 0.151). For one institution, the V12 increases up to 40% for GTV volumes smaller than 1 cm^3^. Nevertheless, the risk for radionecrosis was not affected in those cases. This observation was related to the increase in PTV volume resulting from the anisotropic margin applied to small targets in the OMC, as shown in Figure 5. This increase was driven by the minimal craniocaudal dimension, which was a consequence of the CT slice thickness.

The analysis of the plans consisted of reviewing PCI, GI, V12, and the other indices in that order. Two OMC plans (from institutions B and C) showed similar degradation in GMC plans for the PCI and V12 in some lesions. This was related to the effect of dose cluster formation produced by the proximity of diverse metastases, and the higher modulation of the dose due to the presence of 15 and 19 brain metastasis, respectively. These cases were accepted as dose coverage, and V12 was not compromised, as shown in Figure 6 and Tables 3 and 4. An example of this evaluation is shown in Supporting Information.

## 4 DISCUSSION

SIMM‐SRS involves interrelated mechanical, dosimetric, and clinical challenges, which forces this technique to evaluate all possible sources of uncertainty associated with the treatment process. In particular, the displacements produced by rotations/translations during the patient setup are crucial.^40^, ^41^, ^42^ Different studies have demonstrated with mathematical^43^ and computational approaches^20^, ^44^ that rotation/translation and the order performed depend largely on the relative distance of each lesion from the isocenter. For lesions with a distance less than 50 mm, this impact could be up to 2.5 mm, applying 0.5°/0.5 mm displacements^20^ before setup correction by couch and image guidance, and the effect decreases up to 0.5 mm and 0.2° after correction using image guidance.^45^, ^46^ A strong correlation was described between the total number of metastases for the volume of irradiated brain.^47^ In the same study, a strong correlation was outlined between the volume of lesions and the quality indices of treatment plans, suggesting a dependency between the quality of treatment with these parameters. For this reason, this work presents a simplified method for optimized anisotropic margins considering these variables.

This work displays an original method based on regressor strategies, including the simplest linear model, and more complex models such as XGB. The use of grid search techniques helps to improve the hyperparameters, minimizing the risk of overfitting the models. Then following the strategies to prevent overfitting, their implementation is easy, following a user‐friendly method regardless of the physicist's expertise in SRS, to recommend optimized anisotropic margins that consider mechanical, dosimetric, and geometric parameters.

In particular, we briefly highlight the pros and cons of the different models based on their assumptions. Through the analysis of correlations between the variables, it is shown that the dataset has no multicollinearity. The values of the margin for one observation do not depend on the value of the dependent variable for another observation (it has independence). The effect of changes in a predictor variable on the response variable is consistent regardless of the values of the other variables (demonstrated by bootstrapping method). Thus, the dataset has additivity. We studied the effect of the models with feature selection, shown in Figure 2, and found that the number of independent variables had an impact on the variance of errors and reduced overfitting (demonstrated by the use of data augmentation), thus the data has no homoscedasticity. Therefore, LM fulfills three of four assumptions and has the lowest MAE score.

The pros for RFR are that the prediction from the individual trees has very low correlations, and the cons are that the input data is not continuous for all variables and the target data is discrete. An advantage of MLP is the robustness of its architecture; however, it showed lower MAE and CVS scores compared with other regressor strategies. Finally, XGB showed the best scores for MAE and CVS, and additionally it assumes that the data may not be complete, and it can handle sparsity (e.g., the input variable of location in the brain or dose cluster formation).

To reduce overfitting, more data were introduced by the Gaussian noise injection. With this method, noise is added to each training input value. This causes the training data to fluctuate during training, making it difficult for the regressor strategy to find a solution that fits precisely to the original training dataset and thereby reduces overfitting.^48^, ^49^ It has a zero mean and a controllable standard deviation, allowing to adjust the intensity of the noise as shown in Figure 3, improving the accuracy and MAE by the standard deviation for a reasonable 2% of the input values. Additionally, the reliability and prediction uncertainty of the model predictions in Figure 3 by the bootstrap distributions were appropriate for median‐unbiased estimators of minimum risk, and no bias was found.

The dosimetric impact of the plans can help to explore different aspects of the comparison between GMC and OMC. D99 and PCI showed that coverage and conformity were not compromised as the mean value is similar. GI showed an improvement in gradient and dose falloff. EI shows that conformity and gradient improved for institutions A and C; however, this was not the case for institution B. This was related to the fact that institution B controlled the level of inhomogeneity inside the target to achieve clinically acceptable indices. Therefore, as the target became smaller with the optimized margins, but the maximum dose inside the target was controlled, the EI scored less. To assess plan complexity, MU was reported. At all three institutions, the MU for OMC was lower than that for GMC.

The results indicate a benefit in reducing the margin to a value that ensures a treatment plan with similar conformity, similar complexity, but steeper gradients (and lower V12, due to the smaller PTV margin), and higher efficiency (only due to steeper gradients) is achievable, taking into consideration the different sources of uncertainty in the dose delivery of linac‐based SRS. Moreover, the multiple margin definitions, shown in Table 1, described in this study provide robustness, due to the similar behavior in dose conformity, gradient, dose falloff, complexity, and V12 in the three institutions, shown in Tables 3 and 4, regardless of the isotropic margin assignment criterion.

Although the optimal margins in this study were evaluated for the DCA technique by SIMM‐SRS, it may also be promising in VMAT, provided the margin‐prediction model can be adapted. Furthermore, given VMAT's higher modulation, margin predictions may require additional constraints that were not modeled in this work (such as mean MLC gap or MLC speed).

Recently, the impact of MLC errors on target coverage and normal brain tissue was shown to be comparable between DCA and VMAT based on dosimetric differences of quality indices for the two techniques.^50^ Thus, future work could consider additional parameters for margin prediction models related to dosimetric errors caused by wrong MLC gaps, speeds, and/or gantry speed. This can be performed through different strategies (such as histogram binning, cross‐validation, or isotonic regression), but would need to be evaluated before clinical use.

It is important to mention that although, assigning a 1 mm isotropic margin may be sufficient in the majority of cases (as the maximum acceptable uncertainty in SRS is 1 mm), since there is no single consensus the overestimation of the margin potentially increases the volume of the brain irradiated. Thus, it is suggested that efforts are made to optimize these margins, without compromising the main objective of the treatment. Nevertheless, the use of PTV margins in multiple brain metastases SRS is controversial and has been criticized due to physical and clinical considerations. Some publications have shown an increased risk of radionecrosis and variability in local control when GTV to PTV margins are increased.^51^, ^52^, ^53^, ^54^ Other studies considered that the target control rate and toxicity to normal tissues are largely unchanged when a 1 mm of PTV margin is applied.^55^, ^56^, ^57^, ^58^, ^59^, ^60^, ^61^ However, there are also reports showing that local control is modality and equipment‐specific,^62^ making generalization difficult regarding margin implications.

Nonetheless, the criteria for establishing arbitrary margins of 1 mm for each lesion may be biased due to the limited number of plans reported and other sources of uncertainty not being considered such as CT slice thickness, diameter of the lesion, width of MLC leaves, intrafractional movements, and image verification on couch rotation. Additionally, this criterion has the disadvantage that volume grows in a cubic relationship for the radius of the lesion so that for moderate‐sized metastases or a high number of brain metastases per plan, the volume to be irradiated is greater and therefore, the volume of healthy tissue irradiated is greater, increasing the risk of radionecrosis.^39^

To the protection of the brain, V12 was improved with OMC compared to GMC, showing statistically significant differences (*p* = 0.045). In particular cases, V12 increased due to the dose cluster formation but they were considered acceptable. Tolerances for the V12 could be between 10 and 15 cm^3^.^25^, ^63^ A study showed a trend of V12 associated with increased toxicity risks, and a high rate (∼50%) of toxicity with V12 >10 cm^3^.^36^, ^64^ However, these reports established values considering mainly SRS plans in machines like Gamma Knife, where the considerations may differ from a linear accelerator. Moreover, one of the radionecrosis models used in this work^39^ can help to understand the radiosensitivity of different brain regions. Nonetheless, a limitation of the study is that is mainly attributed to arteriovenous malformations.^39^

In this study, for the GMC plans, the V12 volumes were lower than the constraint of 10 cm^3^ in most cases (up to 38.20 cm^3^) and all of them were clinically approved to be treated due to the compromise with dose coverage to the lesions. For complex cases, it is remarked some limitations of this approach, such as the potential for increased dose bridging of clustered lesions and the constraint imposed by CT slice thickness, particularly affecting margin definition in the craniocaudal direction. Such limitations may impact clinical decision‐making, potentially affecting the balance between tumor control and the risk of toxicity. However, at the present day, there is a lack of studies that evaluate brain toxicity locally, in adjacent regions to the lesions. In this work, a first approximation of this effect was presented, following the existing model of NTCP. The plans that exceeded the constraint for the brain were related to a higher number of treated lesions (>10 metastases) and the dose cluster formation. Furthermore, it is important to notice that the NTCP model followed in this work considers the probability of oedema and necrosis in the brain including the GTVs.

While the reduction in V12 reached statistical significance, its clinical impact may be modest. Nevertheless, even small reductions in V12 could be clinically relevant in situations where the risk of radionecrosis is a major concern, and thus may still influence clinical decision‐making, particularly in complex cases involving multiple‐metastases.

Additionally, the use of optimized margins did not present a significant increase in dose to the brain, as presented in Table 4 and Figure 6. It is noteworthy that there are many situations in which it will be impossible to have a V12 volume < 10–15 cm^3^ and the solution to reduce the margins is not ideal as the uncertainties involved in the whole process must be considered, therefore a clinically acceptable solution may be to accept a larger V12 or increase the number of fractions. It is important to remark that the architecture of the regressor models designed in this work does not include as input any radionecrosis risk indicator. The risk is estimated a posteriori based on the plan dose calculation using the proposal of optimized margins based on OMC and fulfillment with the dose constraints.

It should be mentioned that a more detailed description of susceptibility to necrosis/oedema in different regions of the brain for brain metastases cases due to necrosis risks could be technology dependent and the risks may vary between institutions in planning/delivering practices. Thus, more research is required regarding the clinical impact of focused dose in brain regions as well as more detailed radiobiological models that have a representation of the dose‐response relationship for high‐dose SRS treatments in a single‐fraction, as well as of global and local tumor control probability models.

## 5 CONCLUSION

The regressor models proposed in this work were adequate to predict anisotropic margins that consider the dosimetric, geometric, and setup uncertainty information in single‐fraction, single‐isocenter multiple‐metastasis radiosurgery planned with dynamic conformal arcs.

This study evaluates the feasibility of implementing anisotropic margins in SRS for multiple brain metastases. While some statistically significant differences were observed in dosimetric metrics such as V12 and gradient index, the primary aim was not to establish superiority over conventional uniform margins criteria, but to explore whether such an approach can be effectively integrated into clinical workflows.

The results suggest that anisotropic margins are a viable option and may offer potential benefits in select clinical scenarios, particularly where brain sparing is a priority. This lays the groundwork for further investigation into their potential advantages in more complex or dose‐sensitive cases.

## AUTHOR CONTRIBUTIONS

**José Alejandro Rojas‐López**: Conceptualization; data curation; formal analysis; investigation; methodology; software; supervision; validation; visualization; writing—original draft and review & editing. **Miguel Ángel Chesta**: Formal analysis; methodology; supervision; validation; review & editing. **Francisco Tamarit**: Investigation; software; validation, review & editing. **Néstor Daniel Vacca Campos**: Resources; validation; review & editing. **Mariana Hernández Bojórquez**: Resources; validation; review & editing. **Maximiliano Musso**: Investigation & methodology. **Alexis Dimitriadis**: Review & Editing. **Carlos Daniel Venencia**: Conceptualization; formal analysis; investigation; methodology; supervision; validation; visualization; review & editing.

## CONFLICT OF INTEREST STATEMENT

The authors declare that they have no known competing financial interests or personal relationships that could have appeared to influence the work reported in this study.

All data supporting the findings of this study are available within the paper and its Supplementary Information.

This research received no specific grant from any funding agency in the public, commercial, or not‐for‐profit sectors.

## DATA AVAILABLITY STATEMENT

Research data are available at https://github.com/alxrojas/Regressor‐Metastases.

## Supporting information



Supporting Information
