# Supplementary material for: Optimizing anisotropic margins in single‐isocenter multiple brain metastases radiosurgery using regressor strategies: A multi‐institutional validation study
Source: J Appl Clin Med Phys. 2025 Aug 31;26(9):e70249. doi: 10.1002/acm2.70249 (PMC12398955; doi:10.1002/acm2.70249)
Supplement: Supplementary file 1 — Supporting Information [file ACM2-26-e70249-s001.pdf]

Regressor strategies used to predict PTV margins on single isocenter multiple brain metastases

Created by M.Sc. José Alejandro Rojas López

Contact: alejandro.rojas.lopez@mi.unc.edu.ar (mailto:alejandro.rojas.lopez@mi.unc.edu.ar)

Credits: To Instituto Zunino for providing the clinical information and the resources.

The use of these code and the dataset has open and closed validation. The clinical use is completely responsability of the final user. The authors recommend to validate the dose distributions and the dosimetric indices and reviewing the plan with the clinical staff of each institution before the final use of it.

More information is described in:

1. https://pubmed.ncbi.nlm.nih.gov/37311445/ (https://pubmed.ncbi.nlm.nih.gov/37311445/)
2. https://pubmed.ncbi.nlm.nih.gov/34062337/ (https://pubmed.ncbi.nlm.nih.gov/34062337/)
3. https://pypi.org/project/dicomhandler/ (https://pypi.org/project/dicomhandler/)

Import libraries

In [1]:

```
import pandas as pd
import numpy as np
import time

import matplotlib.pyplot as plt

from sklearn.model_selection import train_test_split
from sklearn.metrics import mean_absolute_error
from sklearn.metrics import accuracy_score
from sklearn.neural_network import MLPRegressor
from sklearn.ensemble import RandomForestRegressor
from sklearn.preprocessing import MinMaxScaler
from sklearn.model_selection import train_test_split, GridSearchCV, KFold, cross_val
from sklearn.feature_selection import f_regression, mutual_info_regression
from sklearn.linear_model import LinearRegression
from sklearn.linear_model import RidgeCV
from sklearn.datasets import make_regression
from time import time
from sklearn.feature_selection import SelectFromModel

from xgboost import XGBRegressor
import matplotlib.ticker as tck

import warnings

warnings.filterwarnings('ignore')
```

Import the dataset

The dataset is called va.csv and is fed by 360 brain metastases of 55 plans for a single institution. We used data augmentation adding "noise" to the input values to increase the dataset to 2160 brain metastases.

The dataset is divided in a training set (75% of the data: 316 mets) and a closed-validation set (25% of the data: 44 mets)

In [2]:

```
df = pd.read_csv("vb.csv")
dataset = df.values
X = dataset[:, 0:6]
y = dataset[:, 6]
X_train, X_test, y_train, y_test = train_test_split(
    X, y, test_size=0.25, random_state=42
)
```

Normalize the dataset

For analyzing the over- under-fitting, we normalized the dataset as follows:

In [3]:

```
norm = MinMaxScaler().fit(X_train)

X_train_norm = norm.transform(X_train)

X_test_norm = norm.transform(X_test)
```

Feature selection

To prevent over-fitting, we applied feature selection to discard the inputs with no relevant information.

In [4]:

```
mi = mutual_info_regression(X, y)
mi /= (sum(mi))
mi = mi*100

ridge = RidgeCV(alphas=np.logspace(-6, 6, num=5)).fit(X, y)
importance = np.abs(ridge.coef_)
feature_names = ["Total number of mets","Location in brain lobes","GTV volume","GTV
plt.bar(height=mi, x=feature_names)
plt.xticks(rotation=30, ha='right')
plt.title("Feature importances via mutual information")
plt.ylabel("Coefficient [%]")
plt.show()
```

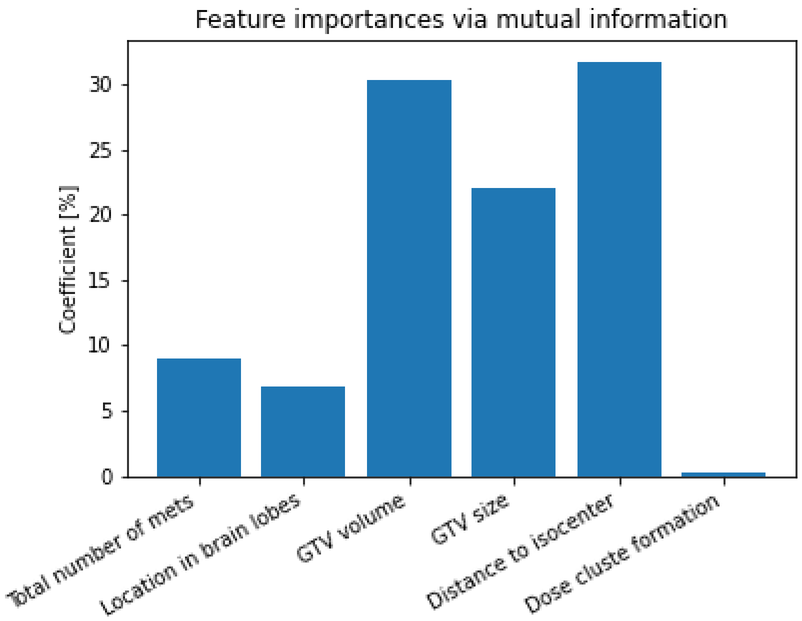

New dataset with feature selection

We deleted dose cluster formation and GTV size inputs in the va_fs.csv file

In [5]:

```
dffs = pd.read_csv("vb_fs.csv")
dataset = dffs.values
Xfs = dataset[:, 0:5]
yfs = dataset[:, 5]
Xfs_train, Xfs_test, yfs_train, yfs_test = train_test_split(
    Xfs, yfs, test_size=0.25, random_state=42
)
```

New dataset without outliers value (<40 mets)

In [6]:

```
df40 = pd.read_csv("vb40.csv")
dataset = df40.values
X40 = dataset[:, 0:6]
y40 = dataset[:, 6]
X40_train, X40_test, y40_train, y40_test = train_test_split(
    X40, y40, test_size=0.25, random_state=42
)
```

Linear model

We create a linear model, reporting CSV, accuracy and MAE.

In [7]:

```
lm = LinearRegression()
lm.fit(X_train, y_train)

predicted_values_lm_train = lm.predict(X_train)
MAE_lm_train = mean_absolute_error(y_train, predicted_values_lm_train)

predicted_values_lm_test = lm.predict(X_test)
MAE_lm_test = mean_absolute_error(y_test, predicted_values_lm_test)

print("Accuracy training MAE: {:.3f}".format(lm.score(X_train, y_train)))
print("LM training MAE : {:.3f}".format(MAE_lm_train))

print("Accuracy test MAE: {:.3f}".format(lm.score(X_test, y_test)))
print("LM validation MAE : {:.3f}".format(MAE_lm_test))

print("CSV for LM: {:.3f}".format(cross_val_score(lm, X_train, y_train,cv=KFold(n_sp
```

```
Accuracy training MAE: 0.598
LM training MAE : 0.076
Accuracy test MAE: 0.600
LM validation MAE : 0.079
CSV for LM: 0.594
```

In [8]:

```
lmfs = LinearRegression()
lmfs.fit(Xfs_train, yfs_train)

predicted_values_lmfs_train = lmfs.predict(Xfs_train)
MAE_lmfs_train = mean_absolute_error(yfs_train, predicted_values_lmfs_train)

predicted_values_lmfs_test = lmfs.predict(Xfs_test)
MAE_lmfs_test = mean_absolute_error(yfs_test, predicted_values_lmfs_test)

print("LM with Feature selection")
print("Accuracy training MAE: {:.3f}".format(lmfs.score(Xfs_train, yfs_train)))
print("LMwFS training MAE : {:.3f}".format(MAE_lmfs_train))

print("Accuracy test MAE: {:.3f}".format(lmfs.score(Xfs_test, yfs_test)))
print("LMwFS validation MAE : {:.3f}".format(MAE_lmfs_test))

print("CSV for LMwFS: {:.3f}".format(cross_val_score(lmfs, Xfs_train, yfs_train,cv=K
```

```
LM with Feature selection
Accuracy training MAE: 0.597
LMwFS training MAE : 0.076
Accuracy test MAE: 0.596
LMwFS validation MAE : 0.080
CSV for LMwFS: 0.594
```

In [9]:

```
lmn = LinearRegression()
lmn.fit(X_train_norm, y_train)

predicted_values_lmn_train = lm.predict(X_train_norm)
MAE_lmn_train = mean_absolute_error(y_train, predicted_values_lmn_train)

predicted_values_lmn_test = lmn.predict(X_test_norm)
MAE_lmn_test = mean_absolute_error(y_test, predicted_values_lmn_test)

print("Accuracy training MAE: {:.3f}".format(lmn.score(X_train_norm, y_train)))
print("LMn training MAE : {:.3f}".format(MAE_lmn_train))

print("Accuracy test MAE: {:.3f}".format(lmn.score(X_test_norm, y_test)))
print("LMn validation MAE : {:.3f}".format(MAE_lmn_test))

print("CSV for LMn: {:.3f}".format(cross_val_score(lmn, X_train_norm, y_train,cv=KFo
```

```
Accuracy training MAE: 0.598
LMn training MAE : 0.290
Accuracy test MAE: 0.600
LMn validation MAE : 0.079
CSV for LMn: 0.594
```

In [10]:

```
lm40 = LinearRegression()
lm40.fit(X40_train, y40_train)

predicted_values_lm40_train = lm40.predict(X40_train)
MAE_lm40_train = mean_absolute_error(y40_train, predicted_values_lm40_train)

predicted_values_lm40_test = lm40.predict(X40_test)
MAE_lm40_test = mean_absolute_error(y40_test, predicted_values_lm40_test)

print("Accuracy training MAE: {:.3f}".format(lm40.score(X40_train, y40_train)))
print("LM40 training MAE : {:.3f}".format(MAE_lm40_train))

print("Accuracy test MAE: {:.3f}".format(lm40.score(X40_test, y40_test)))
print("LM40 validation MAE : {:.3f}".format(MAE_lm40_test))

print("CSV for LM40: {:.3f}".format(cross_val_score(lm40, X40_train, y40_train,cv=KF
```

```
Accuracy training MAE: 0.588
LM40 training MAE : 0.081
Accuracy test MAE: 0.557
LM40 validation MAE : 0.081
CSV for LM40: 0.581
```

Multilayer perceptron (MLP)

We create a multilayer perceptron regressor by grid search strategy, reporting CSV, accuracy, MAE value and the best parameters.

The grid search strategy was based on the following:

In [11]:

```
mlp = MLPRegressor(
    hidden_layer_sizes=100,
    activation="relu",
    random_state=100,
    solver="adam",
    alpha=0.5,
    epsilon=0.00000001,
    max_iter=500,
    max_fun=500,
    learning_rate_init=0.001,
    verbose=False,
).fit(X_train, y_train)
```

In [12]:

```
param_grid = {
    'hidden_layer_sizes': [5,10,20,50,100],
    'learning_rate_init' : [0.001,0.01,0.05,0.1],
    'alpha': [0.0001,0.3,0.5,0.7,0.9]
}

grid_search = GridSearchCV(estimator=MLPRegressor(random_state=0),
param_grid=param_grid,cv=KFold(n_splits=5, shuffle=True, random_state=1));

grid_search.fit(X_train, y_train);
```

In [13]:

```
print(grid_search.best_params_)
```

```
{'alpha': 0.5, 'hidden_layer_sizes': 100, 'learning_rate_init': 0.001}
```

In [14]:

```
predicted_values_mlp_train = mlp.predict(X_train)
MAE_mlp_train = mean_absolute_error(y_train, predicted_values_mlp_train)

predicted_values_mlp_test = mlp.predict(X_test)
MAE_mlp_test = mean_absolute_error(y_test, predicted_values_mlp_test)

print("Accuracy training MAE: {:.3f}".format(mlp.score(X_train, y_train)))
print("MLP training MAE : {:.3f}".format(MAE_mlp_train))

print("Accuracy test MAE: {:.3f}".format(mlp.score(X_test, y_test)))
print("MLP validation MAE : {:.3f}".format(MAE_mlp_test))

print("CSV for MLP: {:.3f}".format(grid_search.best_score_))
```

```
Accuracy training MAE: 0.422
MLP training MAE : 0.103
Accuracy test MAE: 0.494
MLP validation MAE : 0.103
CSV for MLP: 0.625
```

In [15]:

```
mlpfs = MLPRegressor(
    hidden_layer_sizes=100,
    activation="relu",
    random_state=100,
    solver="adam",
    alpha=0.7,
    epsilon=0.00000001,
    max_iter=500,
    max_fun=500,
    learning_rate_init=0.05,
    verbose=False,
).fit(Xfs_train, yfs_train)
```

In [16]:

```
param_grid = {
    'hidden_layer_sizes': [5,10,20,50,100],
    'learning_rate_init' : [0.001,0.01,0.05,0.1],
    'alpha': [0.0001,0.3,0.5,0.7,0.9]
}

grid_search = GridSearchCV(estimator=MLPRegressor(random_state=0),
param_grid=param_grid,cv=KFold(n_splits=5, shuffle=True, random_state=1))

grid_search.fit(Xfs_train, yfs_train)
```

Out[16]:

```
GridSearchCV
estimator: MLPRegressor
MLPRegressor
```

In [17]:

```
print(grid_search.best_params_)
```

```
{'alpha': 0.7, 'hidden_layer_sizes': 100, 'learning_rate_init': 0.05}
```

In [18]:

```
predicted_values_mlpfs_train = mlpfs.predict(Xfs_train)
MAE_mlpfs_train = mean_absolute_error(yfs_train, predicted_values_mlpfs_train)

predicted_values_mlpfs_test = mlpfs.predict(Xfs_test)
MAE_mlpfs_test = mean_absolute_error(yfs_test, predicted_values_mlpfs_test)

print("MLP with Feature selection")

print("Accuracy training MAE: {:.3f}".format(mlpfs.score(Xfs_train, yfs_train)))
print("MLPwFS training MAE : {:.3f}".format(MAE_mlpfs_train))

print("Accuracy test MAE: {:.3f}".format(mlpfs.score(Xfs_test, yfs_test)))
print("MLPwFS validation MAE : {:.3f}".format(MAE_mlpfs_test))

print("CSV for MLPwFS: {:.3f}".format(grid_search.best_score_))
```

```
MLP with Feature selection
Accuracy training MAE: 0.578
MLPwFS training MAE : 0.082
Accuracy test MAE: 0.599
MLPwFS validation MAE : 0.084
CSV for MLPwFS: 0.621
```

In [19]:

```
mlpn = MLPRegressor(
    hidden_layer_sizes=100,
    activation="relu",
    random_state=100,
    solver="adam",
    alpha=0.0001,
    epsilon=0.00000001,
    max_iter=500,
    max_fun=500,
    learning_rate_init=0.01,
    verbose=False,
).fit(X_train_norm, y_train)
```

In [20]:

```
param_grid = {
    'hidden_layer_sizes': [5,10,20,50,100],
    'learning_rate_init' : [0.001,0.01,0.05,0.1],
    'alpha': [0.0001,0.3,0.5,0.7,0.9]
}

grid_search = GridSearchCV(estimator=MLPRegressor(random_state=0),
param_grid=param_grid,cv=KFold(n_splits=5, shuffle=True, random_state=1))

grid_search.fit(X_train_norm, y_train)
```

Out[20]:

```
▸ GridSearchCV
▸ estimator: MLPRegressor
  ▸ MLPRegressor
```

In [21]:

```
print(grid_search.best_params_)
```

```
{'alpha': 0.0001, 'hidden_layer_sizes': 100, 'learning_rate_init': 0.0
1}
```

In [22]:

```
predicted_values_mlpn_train = mlpn.predict(X_train_norm)
MAE_mlpn_train = mean_absolute_error(y_train, predicted_values_mlpn_train)

predicted_values_mlpn_test = mlpn.predict(X_test_norm)
MAE_mlpn_test = mean_absolute_error(y_test, predicted_values_mlpn_test)

print("MLP with normalization")

print("Accuracy training MAE: {:.3f}".format(mlpn.score(X_train_norm, y_train)))
print("MLPn training MAE : {:.3f}".format(MAE_mlpn_train))

print("Accuracy test MAE: {:.3f}".format(mlpn.score(X_test_norm, y_test)))
print("MLPn validation MAE : {:.3f}".format(MAE_mlpn_test))

print("CSV for MLPn: {:.3f}".format(grid_search.best_score_))
```

```
MLP with normalization
Accuracy training MAE: 0.652
MLPn training MAE : 0.070
Accuracy test MAE: 0.648
MLPn validation MAE : 0.075
CSV for MLPn: 0.625
```

In [23]:

```
mlp40 = MLPRegressor(
    hidden_layer_sizes=100,
    activation="relu",
    random_state=100,
    solver="adam",
    alpha=0.9,
    epsilon=0.00000001,
    max_iter=500,
    max_fun=500,
    learning_rate_init=0.05,
    verbose=False,
).fit(X40_train, y40_train)
```

In [24]:

```
param_grid = {
    'hidden_layer_sizes': [5,10,20,50,100],
    'learning_rate_init' : [0.001,0.01,0.05,0.1],
    'alpha': [0.0001,0.3,0.5,0.7,0.9]
}

grid_search = GridSearchCV(estimator=MLPRegressor(random_state=0),
param_grid=param_grid,cv=KFold(n_splits=5, shuffle=True, random_state=1))

grid_search.fit(X40_train, y40_train)
```

Out[24]:

```
GridSearchCV
estimator: MLPRegressor
MLPRegressor
```

In [25]:

```
print(grid_search.best_params_)
```

```
{'alpha': 0.9, 'hidden_layer_sizes': 100, 'learning_rate_init': 0.05}
```

In [26]:

```
predicted_values_mlp40_train = mlp40.predict(X40_train)
MAE_mlp40_train = mean_absolute_error(y40_train, predicted_values_mlp40_train)

predicted_values_mlp40_test = mlp40.predict(X40_test)
MAE_mlp40_test = mean_absolute_error(y40_test, predicted_values_mlp40_test)

print("MLP <40 mets")

print("Accuracy training MAE: {:.3f}".format(mlp40.score(X40_train, y40_train)))
print("MLP40 training MAE : {:.3f}".format(MAE_mlp40_train))

print("Accuracy test MAE: {:.3f}".format(mlp40.score(X40_test, y40_test)))
print("MLP40 validation MAE : {:.3f}".format(MAE_mlp40_test))

print("CSV for MLP40: {:.3f}".format(grid_search.best_score_))
```

```
MLP <40 mets
Accuracy training MAE: 0.616
MLP40 training MAE : 0.077
Accuracy test MAE: 0.569
MLP40 validation MAE : 0.080
CSV for MLP40: 0.608
```

Random Forest Regressor (RFR)

We create a random forest regressor by grid search strategy, reporting CSV, accuracy, MAE value and the best parameters.

The grid search strategy was based on the following:

In [27]:

```
rfr = RandomForestRegressor(
    n_estimators=500,
    criterion="absolute_error",
    max_depth=8,
    max_features = 5,
    min_samples_leaf=1,
    min_samples_split=2)
rfr.fit(X_train, y_train)
```

Out[27]:

```
RandomForestRegressor

RandomForestRegressor(criterion='absolute_error', max_depth=8, max_fe
atures=5,
                      n_estimators=500)
```

In [28]:

```
param_grid = {
    'n_estimators' : [1,10,50,100,500],
    'max_features': [1,3,5,8],
    'max_depth': [1,3,5,8],
    'min_samples_leaf': [1,2,3],
    'min_samples_split': [2,3,4]}

grid_search = GridSearchCV(estimator=RandomForestRegressor(random_state=0),
param_grid=param_grid,cv=KFold(n_splits=5, shuffle=True, random_state=1))

grid_search.fit(X_train, y_train)
```

Out[28]:

```
GridSearchCV
estimator: RandomForestRegressor
    RandomForestRegressor
```

In [29]:

```
print(grid_search.best_params_)
```

```
{'max_depth': 8, 'max_features': 5, 'min_samples_leaf': 1, 'min_sample
s_split': 2, 'n_estimators': 500}
```

In [30]:

```
predicted_values_rfr_train = rfr.predict(X_train)
MAE_rfr_train = mean_absolute_error(y_train, predicted_values_rfr_train)

predicted_values_rfr_test = rfr.predict(X_test)
MAE_rfr_test = mean_absolute_error(y_test, predicted_values_rfr_test)

print("Accuracy training MAE: {:.3f}".format(rfr.score(X_train, y_train)))
print("RFR training MAE : {:.3f}".format(MAE_rfr_train))

print("Accuracy test MAE: {:.3f}".format(rfr.score(X_test, y_test)))
print("RFR validation MAE : {:.3f}".format(MAE_rfr_test))

print("CSV for RFR: {:.3f}".format(grid_search.best_score_))
```

```
Accuracy training MAE: 0.839
RFR training MAE : 0.042
Accuracy test MAE: 0.775
RFR validation MAE : 0.052
CSV for RFR: 0.875
```

In [31]:

```
rfrfs = RandomForestRegressor(
    n_estimators=500,
    criterion="absolute_error",
    max_depth=8,
    max_features = 5,
    min_samples_leaf=1,
    min_samples_split=2)
rfrfs.fit(Xfs_train, yfs_train)
```

Out[31]:

```
RandomForestRegressor

RandomForestRegressor(criterion='absolute_error', max_depth=8, max_fe
atures=5,
                      n_estimators=500)
```

In [32]:

```
param_grid = {
    'n_estimators' : [1,10,50,100,500],
    'max_features': [1,3,5,8],
    'max_depth': [1,3,5,8],
    'min_samples_leaf': [1,2,3],
    'min_samples_split': [2,3,4]}

grid_search = GridSearchCV(estimator=RandomForestRegressor(random_state=0),
param_grid=param_grid,cv=KFold(n_splits=5, shuffle=True, random_state=1))

grid_search.fit(Xfs_train, yfs_train)
```

Out[32]:

```
▸ GridSearchCV
▸ estimator: RandomForestRegressor
    ▸ RandomForestRegressor
```

In [33]:

```
print(grid_search.best_params_)
```

```
{'max_depth': 8, 'max_features': 5, 'min_samples_leaf': 1, 'min_sample
s_split': 2, 'n_estimators': 500}
```

In [34]:

```
predicted_values_rfrfs_train = rfrfs.predict(Xfs_train)
MAE_rfrfs_train = mean_absolute_error(yfs_train, predicted_values_rfrfs_train)

predicted_values_rfrfs_test = rfrfs.predict(Xfs_test)
MAE_rfrfs_test = mean_absolute_error(yfs_test, predicted_values_rfrfs_test)

print("Accuracy training MAE: {:.3f}".format(rfrfs.score(Xfs_train, yfs_train)))
print("RFRwFS training MAE : {:.3f}".format(MAE_rfrfs_train))

print("Accuracy test MAE: {:.3f}".format(rfrfs.score(Xfs_test, yfs_test)))
print("RFRwFS validation MAE : {:.3f}".format(MAE_rfrfs_test))

print("CSV for RFRwFS: {:.3f}".format(grid_search.best_score_))
```

```
Accuracy training MAE: 0.838
RFRwFS training MAE : 0.042
Accuracy test MAE: 0.780
RFRwFS validation MAE : 0.051
CSV for RFRwFS: 0.870
```

In [39]:

```
rfrn = RandomForestRegressor(
    n_estimators=500,
    criterion="absolute_error",
    max_depth=8,
    max_features = 5,
    min_samples_leaf=1,
    min_samples_split=2)
rfr.fit(X_train_norm, y_train)
```

Out[39]:

```
RandomForestRegressor

RandomForestRegressor(criterion='absolute_error', max_depth=8, max_fe
atures=5,
                      n_estimators=500)
```

In [36]:

```
param_grid = {
    'n_estimators' : [1,10,50,100,500],
    'max_features': [1,3,5,8],
    'max_depth': [1,3,5,8],
    'min_samples_leaf': [1,2,3],
    'min_samples_split': [2,3,4]}

grid_search = GridSearchCV(estimator=RandomForestRegressor(random_state=0),
param_grid=param_grid,cv=KFold(n_splits=5, shuffle=True, random_state=1))

grid_search.fit(X_train_norm, y_train)
```

Out[36]:

```
GridSearchCV
estimator: RandomForestRegressor
RandomForestRegressor
```

In [37]:

```
print(grid_search.best_params_)
```

```
{'max_depth': 8, 'max_features': 5, 'min_samples_leaf': 1, 'min_sample
s_split': 2, 'n_estimators': 500}
```

In [43]:

```
predicted_values_rfrn_train = rfrn.predict(X_train_norm)
MAE_rfrn_train = mean_absolute_error(y_train, predicted_values_rfrn_train)

predicted_values_rfrn_test = rfrn.predict(X_test)
MAE_rfrn_test = mean_absolute_error(y_test, predicted_values_rfrn_test)

print("Accuracy training MAE: {:.3f}".format(rfrn.score(X_train_norm, y_train)))
print("RFRn training MAE : {:.3f}".format(MAE_rfrn_train))

print("Accuracy test MAE: {:.3f}".format(rfrn.score(X_test_norm, y_test)))
print("RFRn validation MAE : {:.3f}".format(MAE_rfrn_test))

print("CSV for RFRn: {:.3f}".format(grid_search.best_score_))
```

```
Accuracy training MAE: 0.839
RFRn training MAE : 0.042
Accuracy test MAE: 0.776
RFRn validation MAE : 0.249
CSV for RFRn: 0.876
```

In [44]:

```
rfr40 = RandomForestRegressor(
    n_estimators=100,
    criterion="absolute_error",
    max_depth=8,
    max_features = 5,
    min_samples_leaf=1,
    min_samples_split=2)
rfr40.fit(X40_train, y40_train)
```

Out[44]:

```
RandomForestRegressor

RandomForestRegressor(criterion='absolute_error', max_depth=8, max_fe
atures=5)
```

In [45]:

```
param_grid = {
    'n_estimators' : [1,10,50,100,500],
    'max_features': [1,3,5,8],
    'max_depth': [1,3,5,8],
    'min_samples_leaf': [1,2,3],
    'min_samples_split': [2,3,4]}

grid_search = GridSearchCV(estimator=RandomForestRegressor(random_state=0),
param_grid=param_grid,cv=KFold(n_splits=5, shuffle=True, random_state=1))

grid_search.fit(X40_train, y40_train)
```

Out[45]:

```
GridSearchCV
estimator: RandomForestRegressor
RandomForestRegressor
```

In [46]:

```
print(grid_search.best_params_)
```

```
{'max_depth': 8, 'max_features': 5, 'min_samples_leaf': 1, 'min_sample
s_split': 2, 'n_estimators': 500}
```

In [47]:

```
predicted_values_rfr40_train = rfrn.predict(X40_train)
MAE_rfr40_train = mean_absolute_error(y40_train, predicted_values_rfr40_train)

predicted_values_rfr40_test = rfr40.predict(X40_test)
MAE_rfr40_test = mean_absolute_error(y40_test, predicted_values_rfr40_test)

print("Accuracy training MAE: {:.3f}".format(rfr40.score(X40_train, y40_train)))
print("RFR40 training MAE : {:.3f}".format(MAE_rfr40_train))

print("Accuracy test MAE: {:.3f}".format(rfr40.score(X40_test, y40_test)))
print("RFR40 validation MAE : {:.3f}".format(MAE_rfr40_test))

print("CSV for RFR40: {:.3f}".format(grid_search.best_score_))
```

```
Accuracy training MAE: 0.843
RFR40 training MAE : 0.250
Accuracy test MAE: 0.800
RFR40 validation MAE : 0.050
CSV for RFR40: 0.876
```

XGBoost Regressor (XBG)

We create a XGBoost regressor by grid search strategy, reporting CSV, accuracy, MAE value and the best parameters.

The grid search strategy was based on the following:

In [48]:

```
xgb = XGBRegressor(
    random_state=1,
    booster="gbtree",
    validate_parameters=False,
    learning_rate=0.3,
    sampling_method="uniform",
    max_depth=8,
    min_child_weight=3,
    subsample=1
).fit(X_train, y_train, verbose=True)
```

In [49]:

```
param_grid = {
    'learning_rate' : [0.001,0.01,0.05,0.1,0.3,0.5],
    'min_child_weight': [1,2,3,4,5,6,7,8],
    'max_depth': [1,2,3,4,5,6,7,8]}

grid_search = GridSearchCV(estimator=XGBRegressor(random_state=0),
param_grid=param_grid,cv=KFold(n_splits=5, shuffle=True, random_state=1))

grid_search.fit(X_train, y_train)
```

Out[49]:

```
▸ GridSearchCV
▸ estimator: XGBRegressor
    ▸ XGBRegressor
```

In [50]:

```
print(grid_search.best_params_)
```

```
{'learning_rate': 0.3, 'max_depth': 8, 'min_child_weight': 3}
```

In [52]:

```
predicted_values_xgb_train = xgb.predict(X_train)
MAE_xgb_train = mean_absolute_error(y_train, predicted_values_xgb_train)

predicted_values_xgb_test = xgb.predict(X_test)
MAE_xgb_test = mean_absolute_error(y_test, predicted_values_xgb_test)

print("Accuracy training MAE: {:.3f}".format(xgb.score(X_train, y_train)))
print("XGB training MAE : {:.3f}".format(MAE_xgb_train))

print("Accuracy test MAE: {:.3f}".format(xgb.score(X_test, y_test)))
print("XGB validation MAE : {:.3f}".format(MAE_xgb_test))

print("CSV for XGB: {:.3f}".format(grid_search.best_score_))
```

```
Accuracy training MAE: 1.000
XGB training MAE : 0.002
Accuracy test MAE: 0.982
XGB validation MAE : 0.011
CSV for XGB: 0.953
```

In [53]:

```
xgbfs = XGBRegressor(
    random_state=1,
    booster="gbtree",
    validate_parameters=False,
    learning_rate=0.3,
    sampling_method="uniform",
    max_depth=7,
    min_child_weight=1,
    subsample=1
).fit(Xfs_train, yfs_train, verbose=True)
```

In [54]:

```
param_grid = {
    'learning_rate' : [0.001,0.01,0.05,0.1,0.3,0.5],
    'min_child_weight': [1,2,3,4,5,6,7,8],
    'max_depth': [1,2,3,4,5,6,7,8]}

grid_search = GridSearchCV(estimator=XGBRegressor(random_state=0),
param_grid=param_grid,cv=KFold(n_splits=5, shuffle=True, random_state=1))

grid_search.fit(Xfs_train, yfs_train)
```

Out[54]:

```
GridSearchCV
estimator: XGBRegressor
XGBRegressor
```

In [55]:

```
print(grid_search.best_params_)
```

```
{'learning_rate': 0.3, 'max_depth': 7, 'min_child_weight': 1}
```

In [56]:

```
predicted_values_xgbfs_train = xgbfs.predict(Xfs_train)
MAE_xgbfs_train = mean_absolute_error(yfs_train, predicted_values_xgbfs_train)

predicted_values_xgbfs_test = xgbfs.predict(Xfs_test)
MAE_xgbfs_test = mean_absolute_error(yfs_test, predicted_values_xgbfs_test)

print("Accuracy training MAE: {:.3f}".format(xgbfs.score(Xfs_train, yfs_train)))
print("XGBwFS training MAE : {:.3f}".format(MAE_xgbfs_train))

print("Accuracy test MAE: {:.3f}".format(xgbfs.score(Xfs_test, yfs_test)))
print("XGBwFS validation MAE : {:.3f}".format(MAE_xgbfs_test))

print("CSV for XGBwFS: {:.3f}".format(grid_search.best_score_))
```

```
Accuracy training MAE: 1.000
XGBwFS training MAE : 0.001
Accuracy test MAE: 0.973
XGBwFS validation MAE : 0.013
CSV for XGBwFS: 0.950
```

In [57]:

```
xgbn = XGBRegressor(
    random_state=1,
    booster="gbtree",
    validate_parameters=False,
    learning_rate=0.3,
    sampling_method="uniform",
    max_depth=8,
    min_child_weight=3,
    subsample=1
).fit(X_train_norm, y_train, verbose=True)
```

In [58]:

```
param_grid = {
    'learning_rate' : [0.001,0.01,0.05,0.1,0.3,0.5],
    'min_child_weight': [1,2,3,4,5,6,7,8],
    'max_depth': [1,2,3,4,5,6,7,8]}

grid_search = GridSearchCV(estimator=XGBRegressor(random_state=0),
param_grid=param_grid,cv=KFold(n_splits=5, shuffle=True, random_state=1))

grid_search.fit(X_train_norm, y_train)
```

Out[58]:

```
▸ GridSearchCV
▸ estimator: XGBRegressor
  ▸ XGBRegressor
```

In [59]:

```
print(grid_search.best_params_)
```

```
{'learning_rate': 0.3, 'max_depth': 8, 'min_child_weight': 3}
```

In [61]:

```
predicted_values_xgbn_train = xgbn.predict(X_train_norm)
MAE_xgbn_train = mean_absolute_error(y_train, predicted_values_xgbn_train)

predicted_values_xgbn_test = xgbn.predict(X_test_norm)
MAE_xgbn_test = mean_absolute_error(y_test, predicted_values_xgbn_test)

print("Accuracy training MAE: {:.3f}".format(xgbn.score(X_train_norm, y_train)))
print("XGBn training MAE : {:.3f}".format(MAE_xgbn_train))

print("Accuracy test MAE: {:.3f}".format(xgbn.score(X_test_norm, y_test)))
print("XGBn validation MAE : {:.3f}".format(MAE_xgbn_test))

print("CSV for XGBn: {:.3f}".format(grid_search.best_score_))
```

```
Accuracy training MAE: 1.000
XGBn training MAE : 0.002
Accuracy test MAE: 0.982
XGBn validation MAE : 0.011
CSV for XGBn: 0.953
```

In [62]:

```
xgb40 = XGBRegressor(
    random_state=1,
    booster="gbtree",
    validate_parameters=False,
    learning_rate=0.5,
    sampling_method="uniform",
    max_depth=8,
    min_child_weight=8,
    subsample=1
).fit(X40_train, y40_train, verbose=True)
```

In [63]:

```
param_grid = {
    'learning_rate' : [0.001,0.01,0.05,0.1,0.3,0.5],
    'min_child_weight': [1,2,3,4,5,6,7,8],
    'max_depth': [1,2,3,4,5,6,7,8]}

grid_search = GridSearchCV(estimator=XGBRegressor(random_state=0),
param_grid=param_grid,cv=KFold(n_splits=5, shuffle=True, random_state=1))

grid_search.fit(X40_train, y40_train)
```

Out[63]:

```
GridSearchCV
estimator: XGBRegressor
XGBRegressor
```

In [64]:

```
print(grid_search.best_params_)
```

```
{'learning_rate': 0.5, 'max_depth': 8, 'min_child_weight': 8}
```

In [65]:

```
predicted_values_xgb40_train = xgb40.predict(X40_train)
MAE_xgb40_train = mean_absolute_error(y40_train, predicted_values_xgb40_train)

predicted_values_xgb40_test = xgbn.predict(X40_test)
MAE_xgb40_test = mean_absolute_error(y40_test, predicted_values_xgb40_test)

print("Accuracy training MAE: {:.3f}".format(xgb40.score(X40_train, y40_train)))
print("XGB40 training MAE : {:.3f}".format(MAE_xgb40_train))

print("Accuracy test MAE: {:.3f}".format(xgb40.score(X40_test, y40_test)))
print("XGB40 validation MAE : {:.3f}".format(MAE_xgb40_test))

print("CSV for XGB40: {:.3f}".format(grid_search.best_score_))
```

```
Accuracy training MAE: 0.999
XGB40 training MAE : 0.003
Accuracy test MAE: 0.953
XGB40 validation MAE : 0.279
CSV for XGB40: 0.959
```

In [66]:

```
fig = plt.figure()
f, axes = plt.subplots(nrows = 2, ncols = 6, sharex=True, sharey = True, gridspec_kw
plt.subplots_adjust(wspace=0, hspace=0)
#plt.yticks(fontsize=20)
#plt.xticks(fontsize=20)

#fig.suptitle('Sharing x per column, y per row')
axes[0][0].scatter(y_train, mlp.fit(X_train, y_train).predict(X_train))
axes[0][0].set_ylabel("Predicted margin [mm]", fontsize=20)
axes[0][0].set_title("MLP, training", fontsize=20)
_ = axes[0][0].plot(y, y, color="orange")
axes[0][0].text(0.6, 1.05, r'Accuracy 42.2%', fontsize=15)
axes[0][0].text(0.6, 1.00, r'MAE 0.103', fontsize=15)
axes[0][0].text(0.6, 0.95, r'CVS 0.63', fontsize=15)
axes[0][0].tick_params(labelsize=20)

axes[1][0].scatter(y_test, mlp.fit(X_train, y_train).predict(X_test))
#axes[1][0].set_xlabel("Expected margin [mm]", fontsize=20)
axes[1][0].set_ylabel("Predicted margin [mm]", fontsize=20)
axes[1][0].set_title("MLP, closed validation", fontsize=20)
axes[1][0].text(0.6, 1.05, r'Accuracy 49.4%', fontsize=15)
axes[1][0].text(0.6, 1.00, r'MAE 0.103', fontsize=15)
axes[1][0].tick_params(labelsize=20)
_ = axes[1][0].plot(y, y, color="orange")

axes[0][1].scatter(y_train, rfr.fit(X_train, y_train).predict(X_train))
#axes[0][1].set_ylabel("Predicted margin [mm]")
axes[0][1].set_title("RFR, training", fontsize=20)
axes[0][1].text(0.6, 1.05, r'Accuracy 84.0%', fontsize=15)
axes[0][1].text(0.6, 1.00, r'MAE 0.042', fontsize=15)
axes[0][1].text(0.6, 0.95, r'CVS 0.88', fontsize=15)
_ = axes[0][1].plot(y, y, color="orange")

axes[1][1].scatter(y_test, rfr.fit(X_train, y_train).predict(X_test))
#axes[1][1].set_xlabel("Expected margin [mm]", fontsize=20)
#axes[1][1].set_ylabel("Predicted margin [mm]")
axes[1][1].set_title("RFR, closed validation", fontsize=20)
axes[1][1].text(0.6, 1.05, r'Accuracy 77.8%', fontsize=15)
axes[1][1].text(0.6, 1.00, r'MAE 0.051', fontsize=15)
axes[1][1].tick_params(labelsize=20)
_ = axes[1][1].plot(y, y, color="orange")

axes[0][2].scatter(yfs_train, rfrfs.fit(Xfs_train, yfs_train).predict(Xfs_train))
#axes[0][2].set_ylabel("Predicted margin [mm]")
axes[0][2].set_title("RFRwFS, training", fontsize=20)
axes[0][2].text(0.6, 1.05, r'Accuracy 100.0%', fontsize=15)
axes[0][2].text(0.6, 1.00, r'MAE 0.001', fontsize=15)
axes[0][2].text(0.6, 0.95, r'CVS 0.95', fontsize=15)
_ = axes[0][2].plot(y, y, color="orange")

axes[1][2].scatter(yfs_test, rfr.fit(Xfs_train, yfs_train).predict(Xfs_test))
axes[1][2].set_xlabel("Expected margin [mm]", fontsize=20)
#axes[1][2].set_ylabel("Predicted margin [mm]")
axes[1][2].set_title("RFRwFS, closed validation", fontsize=20)
axes[1][2].text(0.6, 1.05, r'Accuracy 97.3%', fontsize=15)
axes[1][2].text(0.6, 1.00, r'MAE 0.013', fontsize=15)
axes[1][2].tick_params(labelsize=20)
_ = axes[1][2].plot(y, y, color="orange")
```

```
axes[0][3].scatter(y_train, xgb.fit(X_train, y_train).predict(X_train))
#axes[0][3].set_ylabel("Predicted margin [mm]")
axes[0][3].set_title("XGB, training", fontsize=20)
axes[0][3].text(0.6, 1.05, r'Accuracy 100.0%', fontsize=15)
axes[0][3].text(0.6, 1.00, r'MAE 0.002', fontsize=15)
axes[0][3].text(0.6, 0.95, r'CVS 0.95', fontsize=15)
_ = axes[0][3].plot(y, y, color="orange")

axes[1][3].scatter(y_test, xgb.fit(X_train, y_train).predict(X_test))
#axes[1][3].set_xlabel("Expected margin [mm]", fontsize=20)
#axes[1][3].set_ylabel("Calculated margin [mm]")
axes[1][3].set_title("XGB, closed validation", fontsize=20)
axes[1][3].text(0.6, 1.05, r'Accuracy 98.2%', fontsize=15)
axes[1][3].text(0.6, 1.00, r'MAE 0.011', fontsize=15)
axes[1][3].tick_params(labelsize=20)
_ = axes[1][3].plot(y, y, color="orange")

axes[0][4].scatter(yfs_train, xgbfs.fit(Xfs_train, yfs_train).predict(Xfs_train))
#axes[0][4].set_ylabel("Predicted margin [mm]")
axes[0][4].set_title("XGBwFS, training", fontsize=20)
axes[0][4].text(0.6, 1.05, r'Accuracy 98.1%', fontsize=15)
axes[0][4].text(0.6, 1.00, r'MAE 0.015', fontsize=15)
axes[0][4].text(0.6, 0.95, r'CVS 0.83', fontsize=15)
_ = axes[0][4].plot(y, y, color="orange")

axes[1][4].scatter(yfs_test, xgbfs.fit(Xfs_train, yfs_train).predict(Xfs_test))
#axes[1][4].set_xlabel("Expected margin [mm]", fontsize=20)
#axes[1][4].set_ylabel("Calculated margin [mm]")
axes[1][4].set_title("XGBwFS, closed validation", fontsize=20)
axes[1][4].text(0.6, 1.05, r'Accuracy 82.6%', fontsize=15)
axes[1][4].text(0.6, 1.00, r'MAE 0.048', fontsize=15)
axes[1][4].tick_params(labelsize=20)
_ = axes[1][4].plot(y, y, color="orange")

axes[0][5].scatter(y_train, lm.fit(X_train, y_train).predict(X_train))
#axes[0][5].set_ylabel("Predicted margin [mm]")
axes[0][5].set_title("LM, training", fontsize=20)
axes[0][5].text(0.6, 1.05, r'Accuracy 59.8%', fontsize=15)
axes[0][5].text(0.6, 1.00, r'MAE 0.076', fontsize=15)
axes[0][5].text(0.6, 0.95, r'CVS 0.59', fontsize=15)
_ = axes[0][5].plot(y, y, color="orange")

axes[1][5].scatter(y_test, lm.fit(X_train, y_train).predict(X_test))
#axes[1][5].set_xlabel("Expected margin [mm]", fontsize=20)
#axes[1][5].set_ylabel("Calculated margin [mm]")
axes[1][5].set_title("LM, closed validation", fontsize=20)
axes[1][5].text(0.6, 1.05, r'Accuracy 60.0%', fontsize=15)
axes[1][5].text(0.6, 1.00, r'MAE 0.079', fontsize=15)
axes[1][5].tick_params(labelsize=20)
_ = axes[1][5].plot(y, y, color="orange")

for ax in fig.get_axes():
    ax.label_outer()

plt.gcf().set_size_inches(40,12)
plt.savefig("F1.tiff")
```

```
<Figure size 432x288 with 0 Axes>
```

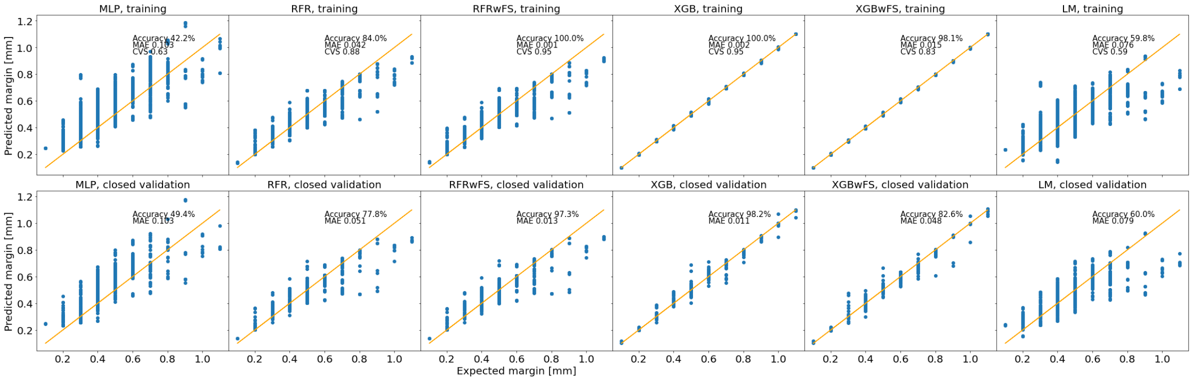

MLP, training
Accuracy 42.2%
MAE 0.103
CVS 0.63
RFR, training
Accuracy 84.0%
MAE 0.042
CVS 0.88
RFRwFS, training
Accuracy 100.0%
MAE 0.001
CVS 0.95
XGB, training
Accuracy 100.0%
MAE 0.002
CVS 0.95
XGBwFS, training
Accuracy 98.1%
MAE 0.015
CVS 0.83
LM, training
Accuracy 59.8%
MAE 0.076
CVS 0.59
MLP, closed validation
Accuracy 49.4%
MAE 0.103
RFR, closed validation
Accuracy 77.8%
MAE 0.051
RFRwFS, closed validation
Accuracy 97.3%
MAE 0.013
XGB, closed validation
Accuracy 98.2%
MAE 0.011
XGBwFS, closed validation
Accuracy 82.6%
MAE 0.048
LM, closed validation
Accuracy 60.0%
MAE 0.079
Predicted margin [mm]
Expected margin [mm]

In [67]:

```
import matplotlib.ticker as tck

fig = plt.figure()
f, axes = plt.subplots(nrows = 2, ncols = 3, sharex=False, sharey = True, gridspec_k
plt.subplots_adjust(wspace=0, hspace=0)
axes[0][0].plot(X_train[:,0], xgb.predict(X_train), 'ko', alpha=0.3)
axes[0][0].plot(X_train[:,0], y_train, 'm*')
axes[0][0].set_ylabel("PTV margin [mm]", fontsize=18)
axes[0][0].set_xlabel("Metastases per plan", fontsize=18)
axes[0][0].tick_params(labelsize=20)
#axes[0][0].set_title("Total number of mets per plan")

axes[1][0].plot(X_train[:,1], xgb.predict(X_train), 'ko', alpha=0.3)
axes[1][0].plot(X_train[:,1], y_train, 'm*')
axes[1][0].set_ylabel("PTV margin [mm]", fontsize=18)
axes[1][0].set_xlabel("Location in the brain", fontsize=18)
axes[1][0].tick_params(labelsize=20)
#axes[1][0].set_title("Location in brain lobes")


axes[0][1].plot(X_train[:,2], xgb.predict(X_train), 'ko', alpha=0.3)
axes[0][1].plot(X_train[:,2], y_train, 'm*')
axes[0][1].set_xlabel("GTV volume [cc]", fontsize=18)
axes[0][1].tick_params(labelsize=20)
#axes[0][1].set_title("GTV volume")


axes[1][1].plot(X_train[:,3], xgb.predict(X_train), 'ko', alpha=0.3)
axes[1][1].plot(X_train[:,3], y_train, 'm*')
axes[1][1].set_xlabel("GTV size [cm]", fontsize=18)
axes[1][1].tick_params(labelsize=20)
#axes[1][1].set_title("GTV size")

axes[0][2].plot(X_train[:,4], xgb.predict(X_train), 'ko', alpha=0.3)
axes[0][2].plot(X_train[:,4], y_train, 'm*')
axes[0][2].set_xlabel("Distance to isocenter [mm]", fontsize=18)
axes[0][2].tick_params(labelsize=20)
#axes[0][2].set_title("Distance to isocenter")

axes[1][2].plot(X_train[:,5], xgb.predict(X_train), 'ko', alpha=0.3, label='XGB mode
axes[1][2].plot(X_train[:,5], y_train, 'm*', label='Training dataset')
axes[1][2].set_xlabel("Dose cluster formation", fontsize=18)
axes[1][2].tick_params(labelsize=20)
#axes[1][2].set_title("Dose cluster formation")

plt.legend(bbox_to_anchor=(1.05, 1.0), loc='upper left', fontsize=18)
plt.tight_layout()

axes[1][2].yaxis.set_minor_locator(tck.AutoMinorLocator())
axes[1][2].xaxis.set_minor_locator(tck.AutoMinorLocator())
axes[1][1].xaxis.set_minor_locator(tck.AutoMinorLocator())
axes[0][1].xaxis.set_minor_locator(tck.AutoMinorLocator())
axes[0][0].xaxis.set_minor_locator(tck.AutoMinorLocator())
axes[0][1].xaxis.set_minor_locator(tck.AutoMinorLocator())
axes[0][2].xaxis.set_minor_locator(tck.AutoMinorLocator())

for ax in fig.get_axes():
    ax.label_outer()
```

```
plt.gcf().set_size_inches(34,12)
plt.savefig("F2.tiff")
```

<Figure size 432x288 with 0 Axes>

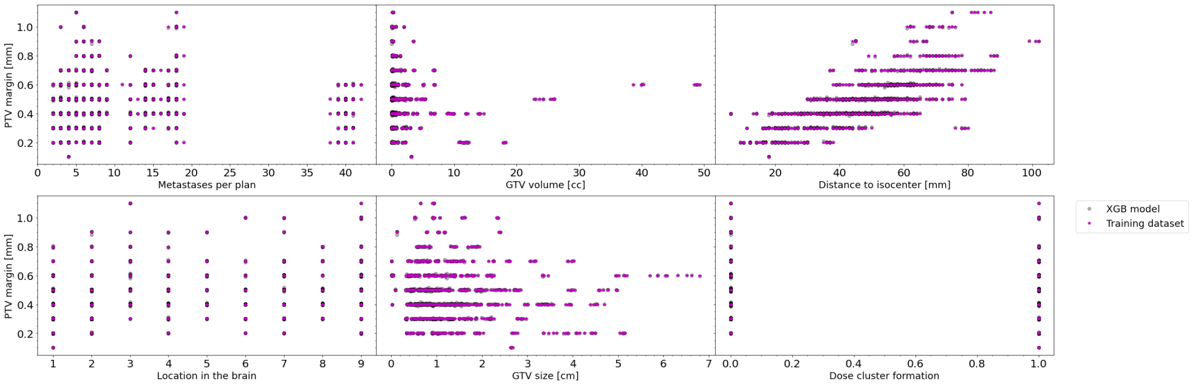

In [ ]:
